# Selected Calcium Silicate-Based Endodontic Cements: From Physicochemical Properties and Molecular Bioactivity to Indication-Specific Clinical Performance

**DOI:** 10.3390/ijms272110042

**Published:** 2026-11-09

**Authors:** Natalia Sędek, Maria Adamska, Kamil Saramowicz, Pengyu Zhao, Konrad Małkiewicz, Wioletta Rozpędek-Kamińska

**Affiliations:** 1 Student Scientific Club of the Department of Orthodontics, Medical University of Lodz, 92-213 Lodz, Poland; natalia.sedek@stud.umed.lodz.pl (N.S.); maria.adamska@stud.umed.lodz.pl (M.A.); 2 Central Teaching Hospital, Medical University of Lodz, 92-213 Lodz, Poland; kamil.saramowicz@stud.umed.lodz.pl; 3 College of Life Science and Technology, Huazhong University of Science and Technology, Wuhan 430074, China; pengyu122601@hust.edu.cn; 4 Key Laboratory of Molecular Biophysics of the Ministry of Education, Wuhan 430074, China; 5 Department of Orthodontics, Medical University of Lodz, 92-213 Lodz, Poland; 6 Medical University of Lodz, 90-419 Lodz, Poland

**Keywords:** calcium silicate-based cements, mineral trioxide aggregate, Biodentine, EndoSequence root repair material, calcium-enriched mixture cement, dental biomaterials, bioactivity, biomineralization, vital pulp therapy, regenerative endodontics

## Abstract

Calcium silicate-based cements are increasingly used in vital pulp therapy, endodontic repair, and regenerative procedures, but their selection remains complicated by differences in formulation, hydration behavior, mechanical maturation, biological activity, and clinical evidence. This review integrates physicochemical, mechanical, biological, and clinical data on mineral trioxide aggregate (MTA), Biodentine, EndoSequence Root Repair Material (ERRM), and calcium-enriched mixture (CEM) cement within a mechanistic-to-clinical framework linking material composition and hydration with ion release, alkalization, interfacial mineralization, cellular responses, and indication-specific performance. MTA remains the reference material with the most mature clinical evidence, although conventional formulations remain limited by prolonged setting, handling difficulties, and discoloration risk. Biodentine combines relatively rapid setting, favorable handling and esthetic characteristics, and pronounced early bioactivity. ERRM provides a convenient premixed formulation and promising mechanical, sealing, and biological performance, whereas CEM cement combines calcium- and phosphate-related bioactivity, antimicrobial activity, and encouraging clinical outcomes, particularly in vital pulp therapy. Across all materials, improvements in individual properties are accompanied by formulation-dependent trade-offs involving solubility, porosity, radiopacity, maturation kinetics, and biological response. Substantial methodological heterogeneity limits direct numerical ranking. Material selection should therefore be indication-specific and integrate material behavior with the maturity of supporting clinical evidence.

## 1. Introduction

One of the most common consequences of deep carious lesions is irreversible pulpitis. The conventional treatment for this condition is root canal therapy, which involves removal of the dental pulp [1].

In recent years, increasing attention has been directed toward biologically based treatment strategies aimed at preserving pulp vitality and promoting endogenous tissue regeneration rather than replacing damaged tissues with inert filling materials. This shift has led to growing interest in bioactive calcium silicate-based cements, which interact with the dentin–pulp complex through calcium and silicate ion release, formation of an alkaline microenvironment, and apatite-forming reactions that support mineralized tissue repair and preservation of pulp vitality [2,3,4].

Depending on the clinical indication and pulpal status, vital pulp therapy (VPT) may involve preserving the existing pulp tissue or selectively removing inflamed tissue, followed by placement of a biologically compatible material to support healing and maintain pulp vitality [2,3].

Mineral Trioxide Aggregate (MTA) was the first hydraulic calcium silicate-based cement introduced into clinical endodontic practice and remains a reference material for vital pulp therapy and endodontic repair procedures. It is composed predominantly of tricalcium and dicalcium silicate phases together with additional mineral components and a radiopacifier, and is characterized by favorable biocompatibility, sealing ability, alkaline activity, and mineralization potential [5,6]. MTA can additionally modulate inflammation- and mineralization-related signaling in human dental pulp stem cells [7].

Clinical studies have reported favorable outcomes for direct pulp capping with selected MTA formulations, although reported success rates vary according to study design, case selection, treatment protocol, and follow-up duration [8,9]. Despite these advantages, conventional MTA formulations retain several limitations, including prolonged setting time, difficult handling, and the risk of tooth discoloration. Consequently, new generations of calcium silicate-based biomaterials have been developed to improve physicochemical performance while preserving biological activity.

Although multiple calcium silicate-based cements have been introduced as alternatives to MTA, their physicochemical characteristics, ion-release profiles, biological interactions with pulp-derived cells, and clinical performance differ substantially. Comparative evaluation of these materials is therefore essential for understanding their mechanisms of action and optimizing their clinical application in regenerative endodontic procedures. The analysis focuses on MTA, Biodentine, ERRM, and CEM cement as selected representatives of calcium silicate-based endodontic materials with distinct formulations, handling characteristics, biological profiles, and levels of clinical evidence. The review is therefore intended as an in-depth comparative evaluation of these four materials rather than as an exhaustive survey of all currently available calcium silicate-based cements.

The distinctive contribution of this review lies in integrating physicochemical, mechanical, biological, and clinical evidence within a single comparative framework. Rather than considering individual material properties in isolation, the analysis links formulation and hydration behavior with ion release and alkalization, interfacial mineralization, cellular responses, and indication-specific clinical performance. This approach also allows formulation-related improvements to be interpreted as interconnected material trade-offs, since gains in setting kinetics, ionic reactivity, handling, or esthetic performance may be accompanied by changes in solubility, mechanical maturation, radiopacity, or biological behavior.

**Figure 1:**
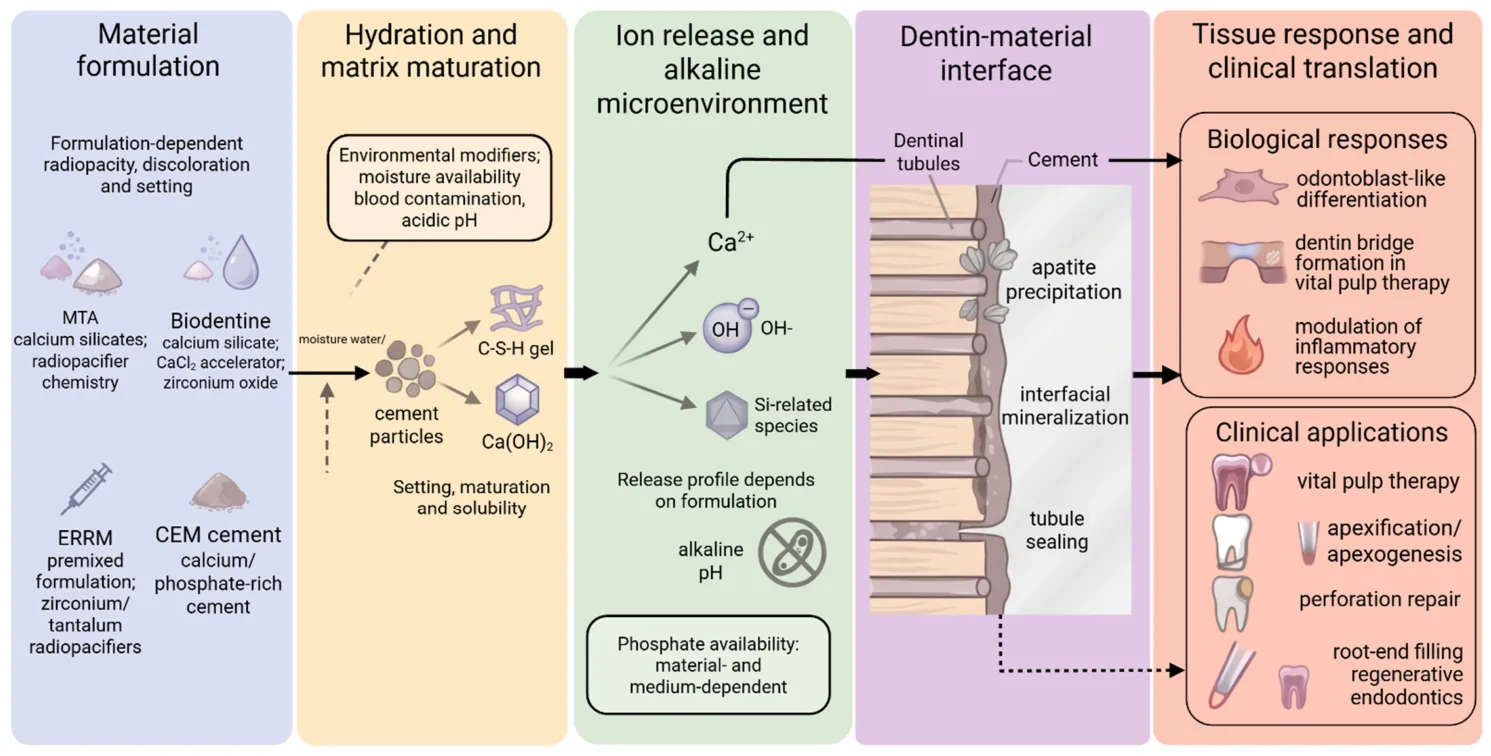
Mechanistic-to-clinical framework linking formulation, hydration and matrix maturation, ion release and the alkaline microenvironment, dentin-material interfacial events, biological responses, and indication-specific clinical applications of calcium silicate-based endodontic cements. Environmental modifiers, including moisture availability, blood contamination, and acidic pH, may influence hydration and maturation, whereas phosphate availability is material- and medium-dependent. The scheme emphasizes that bioactivity does not automatically imply clinical superiority and that material selection should integrate physicochemical behavior, biological response, handling requirements, and maturity of clinical evidence. Created in BioRender. Rozpędek-Kamińska, W. (2026) https://biorender.com/kpdm6q4, accessed on 1 October 2026.

Accordingly, this review provides an integrated comparative analysis of Mineral Trioxide Aggregate (MTA), Biodentine, EndoSequence Root Repair Material (ERRM), and calcium-enriched mixture (CEM) cement, with particular emphasis on how differences in their physicochemical properties and molecular bioactivity translate into mechanical behavior and clinical applicability in vital pulp therapy and endodontic repair. Particular attention is also given to the maturity and translational relevance of the available evidence, allowing material selection to be considered in an indication-specific rather than purely hierarchical manner. The mechanistic-to-clinical framework used to organize this review is summarized in Figure 1.

### Literature Search and Selection

Relevant publications were identified through searches of the PubMed database, last updated on 15 September 2026. Particular emphasis was placed on studies published within the preceding five years, supplemented by earlier foundational and methodologically relevant investigations. Search terms included “mineral trioxide aggregate”, “MTA”, “ProRoot MTA”, “MTA Angelus”, “Biodentine”, “EndoSequence Root Repair Material”, “ERRM”, “TotalFill BC RRM”, “calcium-enriched mixture”, and “CEM cement”, combined with terms related to chemical composition, hydration, physicochemical properties, mechanical performance, biocompatibility, biomineralization, inflammatory responses, antimicrobial activity, and clinical applications in endodontics.

Publications were selected according to their relevance to the four investigated materials, the identification of the tested formulations, and the availability of interpretable experimental or clinical findings. The selection process considered titles and abstracts to establish topical relevance, followed by examination of full-text reports where necessary to verify material composition, experimental methods, outcome definitions, and clinical applicability. Studies were considered for the primary comparative analysis when they investigated at least one of the selected materials and reported relevant physicochemical, mechanical, biological, histological, or clinical outcomes. Both direct comparative investigations and studies evaluating individual materials were eligible. Reports focusing exclusively on unrelated materials or lacking relevant and interpretable outcomes were not considered for the primary comparative synthesis.

Original investigations were prioritized as sources of material-specific quantitative, mechanistic, and clinical evidence. Laboratory and ex vivo studies, animal experiments, human histological investigations, and clinical studies were considered separately to distinguish experimental observations from patient-level treatment outcomes. Systematic reviews and meta-analyses were used to contextualize clinical findings and assess the consistency of evidence across studies, while narrative reviews provided additional mechanistic and historical background. Manufacturer documentation and relevant technical standards were consulted to clarify formulation characteristics and standardized testing requirements.

Studies investigating experimentally modified formulations, precursor materials, composite scaffolds, or root canal sealers were considered when they provided relevant mechanistic or methodological information. However, findings obtained with these preparations were not directly extrapolated to conventional commercial repair cements. Similarly, numerical results from independent studies were interpreted in relation to the specific formulation, specimen preparation, testing conditions, measurement methods, and observation periods. Where methodological information was incomplete, the corresponding findings were interpreted cautiously rather than used to establish direct numerical comparisons or material superiority.

The four materials were selected to represent distinct formulation strategies, physicochemical characteristics, and levels of supporting clinical evidence. MTA was included as the historical reference material with an extensive experimental and clinical evidence base [5,6]. Biodentine represents an accelerated-setting powder–liquid calcium silicate formulation with favorable handling characteristics and an expanding body of clinical evidence [10,11,12,13]. ERRM represents a family of premixed, moisture-activated root repair materials with formulation-dependent physicochemical and biological properties [14,15,16,17]. CEM cement was selected because of its distinct calcium- and phosphate-containing composition and its clinical investigation, particularly in vital pulp therapy [18,19,20]. Together, these materials allow comparison of formulation-related properties, biological responses, and clinical applicability across different endodontic indications.

This review was designed as a focused, non-exhaustive narrative synthesis of representative evidence rather than a systematic assessment of all available calcium silicate-based endodontic materials. The strength and clinical relevance of the available evidence were evaluated qualitatively according to study design, methodological characteristics, sample size, follow-up duration, consistency of findings, and clinical indication, without applying formal risk-of-bias or certainty-of-evidence grading frameworks.

## 2. Calcium Silicate-Based Cements: Biological and Material Foundations

The setting reaction of calcium silicate-based cements (CSCs) is based on hydration. During this process, the main mineral components dissolve and react with water to form hydrated phases. Specifically, the interaction between calcium silicate and water leads to the formation of calcium silicate hydrate (C–S–H) gel and calcium hydroxide, which are responsible for the development of the material’s mechanical strength and bioactivity [2].

CSCs, including Mineral Trioxide Aggregate (MTA), are self-setting hydraulic biomaterials composed primarily of di- and tricalcium silicate phases. Upon mixing with water, these components undergo hydration and form a viscous colloidal calcium silicate hydrate gel that progressively hardens into a dense and structurally stable matrix [10]. The resulting alkaline environment created by calcium hydroxide release contributes not only to antimicrobial activity but also to biological interactions with surrounding tissues.

Tertiary dentin is formed at the dentin–pulp interface as a defensive response to pathological stimuli such as caries or trauma [21]. Depending on the severity of tissue injury, tertiary dentin may be classified as reactionary or reparative dentin. In cases of mild and slowly progressing damage, surviving odontoblasts increase their secretory activity and produce reactionary dentin. In contrast, severe injury results in odontoblast death, and their function is replaced by newly differentiated odontoblast-like cells derived from mesenchymal stem cells of the dental pulp [22].

When pulp exposure occurs as a result of trauma or caries removal, destruction of odontoblasts initiates an inflammatory cascade accompanied by the release of signaling molecules and mediators. These factors stimulate migration, proliferation, and differentiation of dental pulp stem cells into odontoblast-like cells capable of producing a mineralized matrix resembling dentin in both structure and composition. The resulting mineralized tissue bridge protects the pulp and supports tissue healing [21,22,23].

Vital pulp therapy procedures, including direct and indirect pulp capping, are designed to support these endogenous regenerative mechanisms. Indirect pulp capping is performed in cases of non-exposed pulp to stimulate surviving odontoblasts to produce reactionary dentin, whereas direct pulp capping promotes reparative dentinogenesis through differentiation of odontoblast-like cells derived from dental pulp stem cells following pulp exposure [13,21,22].

Evidence for calcium silicate-based cement bioactivity comes from different experimental systems and should be interpreted accordingly. Cell-based studies have reported changes in viability, cytokine expression, and osteogenic or odontogenic differentiation markers following exposure to selected cement formulations [10]. In an ex vivo organotypic model, eluates from set ProRoot MTA, Biodentine, and TotalFill BC RRM Fast Putty influenced mineral deposition in embryonic chick femurs, with responses depending on the material and eluate concentration [24]. These observations support potential biological mechanisms but do not demonstrate regeneration of the human dentin–pulp complex or predict long-term treatment success.

Modern biomaterials used in endodontics, particularly calcium silicate-based cements, have been progressively modified through the incorporation of additional components aimed at improving their physicochemical and clinical performance, including setting time and handling properties. As a result, new generations of CSCs have been developed to overcome limitations associated with earlier materials [25].

These materials are commonly evaluated against mineral trioxide aggregate (MTA), which remains a reference hydraulic calcium silicate cement in endodontic repair [5,6,10,26]. Selected MTA formulations can hydrate and harden in the presence of moisture, but this setting property should not be confused with hemostatic activity. During vital pulp therapy, hemorrhage should be controlled through appropriate clinical measures before material placement, while excessive blood contamination may adversely affect cement maturation and interfacial integrity [5,6,10,27]. The biological compatibility, sealing potential, and mineralization-related activity of selected MTA formulations support their use in vital pulp therapy, perforation repair, apexification, and as barriers in regenerative endodontic procedures [5,6,10].

During the treatment of root perforations, biomaterials are frequently placed in direct contact with blood, which may adversely affect their physical properties, including push-out bond strength [28]. Therefore, an ideal endodontic repair material should demonstrate strong adhesion under blood-contaminated conditions [27] and resistance to dislodging forces associated with prosthetic procedures and functional loading [29].

In addition, an optimal biomaterial should exhibit antibacterial activity, low cytotoxicity, high biocompatibility, osteoinductive potential, and strong sealing ability without inducing tooth discoloration. Favorable handling properties, dimensional stability, moisture tolerance, and cost-effectiveness are also desirable characteristics [2,25].

**Figure 2:**
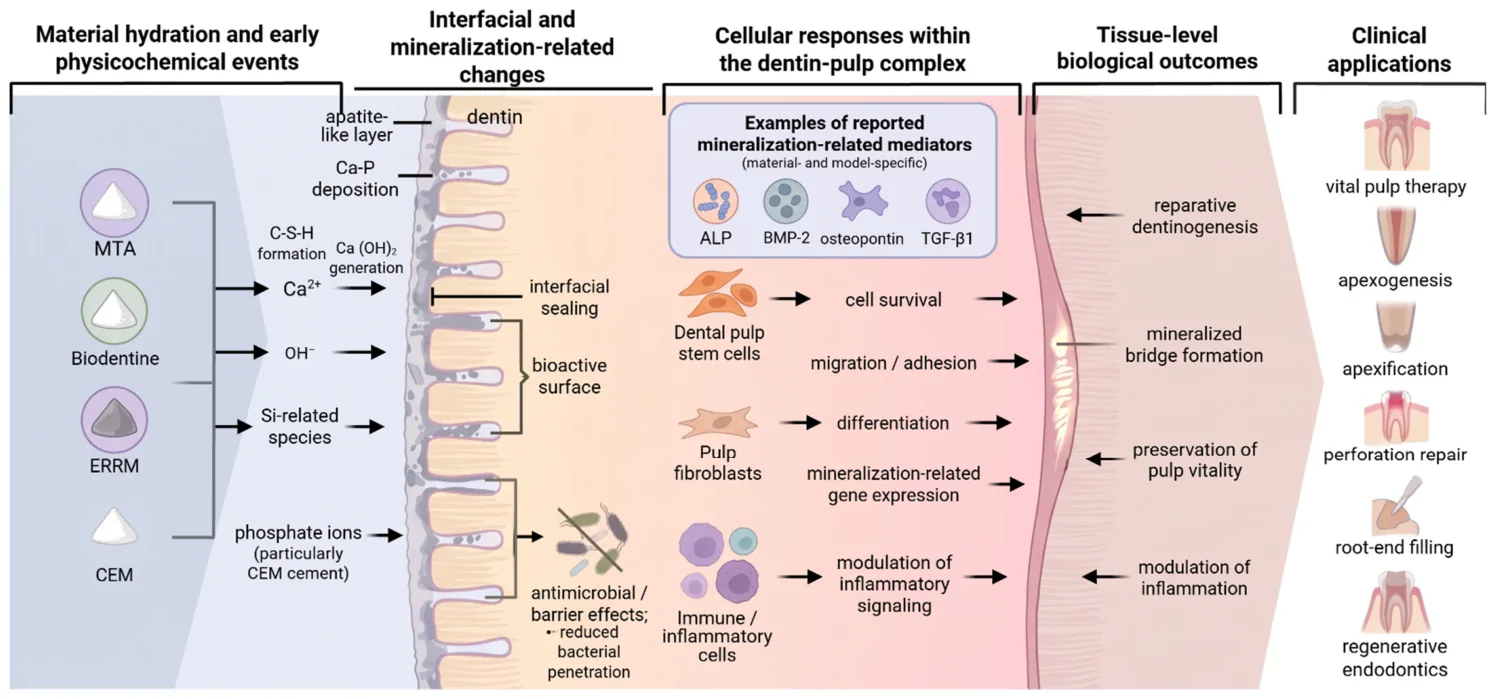
From material hydration to dentin–pulp responses and hard-tissue repair. Calcium silicate-based endodontic cements undergo hydration, ion release, and alkalization, promoting Ca–P deposition, apatite-like interfacial mineralization, sealing, and bioactive surface formation. These events modulate cellular responses within the dentin–pulp complex, including survival, migration, differentiation, inflammatory signaling, and mineralization-related activity. The resulting tissue-level effects may include reparative dentinogenesis, mineralized bridge formation, preservation of pulp vitality, and modulation of inflammation. Reported molecular mediators are material- and model-specific. Created in BioRender. Rozpędek-Kamińska, W. (2026) https://BioRender.com/kpdm6q4, accessed on 1 October 2026.

Ultimately, an ideal endodontic material should promote hard tissue formation and support regenerative processes while establishing a stable biological seal between the material and surrounding tissues [22]. The principal physicochemical, interfacial, cellular, and tissue-level processes linking calcium silicate cement hydration with biological repair are summarized in Figure 2.

## 3. Materials Included in the Comparative Analysis

Throughout this review, “MTA” is used as a generic designation only when referring to findings applicable to MTA-based materials as a group. For formulation-specific numerical or experimental data, the corresponding product name is retained, including ProRoot MTA, MTA Angelus, or MTA Plus.

Similarly, “ERRM” refers to the EndoSequence BC Root Repair Material product family rather than a single formulation. The manufacturer’s product range includes injectable BC RRM, conventional BC RRM Putty, and BC RRM Fast-Set Putty [14,15,16]. The abbreviations ES (regular-setting) and ESF (fast-setting) are used specifically for the formulations designated as such by Khalil and Alharbi [17]. The term “ERRM Fast” follows the nomenclature used by Lee et al. [30] for the tested fast-setting material, whereas “ERRM Putty” refers to the conventional putty formulation investigated by Guo et al. [31]. Product names such as TotalFill BC RRM are retained when explicitly used in the original studies [32]. These labels are not treated as universally interchangeable, and formulation-specific findings are not generalized to the entire ERRM product family.

### 3.1. MTA

Mineral Trioxide Aggregate (MTA) is a calcium silicate-based cement introduced in 1993 that has since gained widespread use in endodontics due to its excellent biocompatibility, favorable sealing ability, and capacity to induce hard tissue formation through calcium ion release [5,33,34]. MTA-based materials have historically been available in gray and white variants, while multiple commercial formulations, including ProRoot MTA and MTA Angelus, differ in composition and physicochemical behavior. White MTA (WMTA) contains lower amounts of aluminum, iron, and magnesium oxides and has been reported to exhibit higher compressive strength than gray MTA (GMTA), whereas GMTA shows a longer setting time and lower radiopacity and pH values [35]. Although the introduction of WMTA reduced the risk of tooth discoloration, this limitation has not been completely eliminated.

Selected MTA repair cements have been used in apexification, apexogenesis, vital pulp therapy, perforation repair, root-end filling, and as coronal barriers in regenerative endodontic procedures [3,5,6]. These repair cements should be distinguished from MTA-containing root canal sealers and modified commercial formulations, such as MTA Repair HP [36], whose experimental findings cannot automatically be generalized to conventional MTA products. Despite this broad spectrum of clinical indications, conventional MTA formulations, particularly ProRoot MTA, are associated with several limitations, including prolonged setting time, difficult handling properties, suboptimal performance when exposed to body fluids, and the potential for coronal discoloration [7,37]. In addition, the relatively high cost of MTA may limit routine clinical use [38].

Conventional MTA formulations, particularly ProRoot MTA, consist primarily of purified Portland cement combined with bismuth oxide as a radiopacifier. In these formulations, the powder contains fine hydrophilic particles that, upon mixing with water, form a colloidal gel containing calcium silicate hydrate and calcium hydroxide, which subsequently hardens into a solid structure. The principal components of purified Portland cement include dicalcium silicate (Ca_2_SiO_4_), tricalcium silicate (Ca_3_SiO_5_), tricalcium aluminate (Ca_3_Al_2_O_6_), calcium sulfate (CaSO_4_), and aluminoferrite (4CaO·Al_2_O_3_·Fe_2_O_3_). Minor constituents include silicon dioxide (SiO_2_), calcium oxide (CaO), magnesium oxide (MgO), potassium sulfate (K_2_SO_4_), and sodium sulfate (Na_2_SO_4_) [10].

### 3.2. Biodentine

Biodentine was introduced in 2011 as a bioactive dentin substitute and has since become one of the most widely investigated alternatives to MTA in vital pulp therapy and endodontic repair procedures. It was developed to preserve the favorable biological properties of calcium silicate-based cements while improving several practical limitations associated with conventional MTA, particularly prolonged setting time, handling difficulties, and the risk of tooth discoloration [10,11,12,13].

It is supplied as a powder contained in a capsule and a liquid provided in a single-dose ampoule. The powder consists primarily of purified tricalcium silicate (3CaO·SiO_2_; 80.1%), calcium carbonate (CaCO_3_; 14.9%) acting as a filler, and zirconium oxide (ZrO_2_) as a radiopacifier, with trace amounts of dicalcium silicate (2CaO·SiO_2_) and calcium oxide (CaO). The liquid phase contains water, calcium chloride (CaCl_2_), which acts as a setting accelerator, and a water-soluble polymer functioning as a plasticizing agent. The presence of CaCl_2_ promotes rapid hydration and early calcium ion release, whereas the polymeric component reduces the amount of water required for mixing and contributes to formation of a relatively dense cement matrix [10,39,40].

Unlike conventional MTA, Biodentine does not contain bismuth oxide and instead uses zirconium oxide to provide radiopacity. This compositional difference reduces the risk of material-associated tooth discoloration and avoids some of the biological and physicochemical concerns linked to bismuth-containing formulations. Biodentine is characterized by pronounced calcium ion release, alkaline pH, and formation of calcium phosphate deposits at the material–dentin interface, which contribute to its biomineralization potential and ability to support reparative dentin formation [41,42,43].

Clinically, Biodentine has been used in direct and indirect pulp capping, pulpotomy, apexogenesis, apexification, perforation repair, root-end filling, and regenerative endodontic procedures [3,11,44]. Its relatively rapid setting and favorable early mechanical properties provide practical advantages in procedures requiring early material stabilization. However, lower radiopacity than MTA, variable long-term solubility, and sensitivity of some mechanical and sealing properties to blood contamination and experimental conditions remain relevant limitations. Thus, Biodentine represents a clinically important MTA alternative whose principal advantages arise from faster maturation, improved handling, and reduced discoloration potential rather than from universal superiority across all material properties.

### 3.3. ERRM

EndoSequence Root Repair Material (ERRM) is a premixed hydraulic calcium silicate-based material available as an injectable paste or putty, including a fast-setting putty formulation. According to the manufacturer’s formulation-specific instructions for use, the listed constituents include calcium silicates, monobasic calcium phosphate, calcium sulfate, zirconium oxide, tantalum pentoxide, and filler agents [14,15,16]. The material requires moisture to initiate hydration and setting, while its handling and setting characteristics depend on the product formulation.

Independent elemental analysis identified Ca, P, Ta, Zr, and Si as major elements in both regular-setting (ES) and fast-setting (ESF) ERRM formulations [17]. However, differences in calcium release, alkalization, and apatite precipitation were observed between these formulations, indicating that similar declared ingredients do not necessarily imply identical physicochemical behavior [17]. The available clinical and preclinical findings are discussed separately in Section 5.1 [45,46].

### 3.4. CEM Cement

Calcium-enriched mixture (CEM) cement is a hydrophilic, tooth-colored biomaterial distinguished by its alkaline pH, bioactivity, and antimicrobial potential [18,19,47]. It has been introduced as an alternative to conventional calcium silicate-based materials, combining the favorable biocompatibility of Mineral Trioxide Aggregate (MTA) with several clinically advantageous features, including shorter setting time, improved handling properties, and a reduced risk of post-treatment tooth discoloration [18,19].

As a member of the calcium silicate cement family, CEM cement exhibits a characteristic physicochemical and biological profile that is closely related to its specific chemical composition [18]. The powder phase contains multiple calcium-containing compounds, including calcium oxide, calcium carbonate, calcium silicate, calcium sulfate, calcium hydroxide, and calcium chloride [48]. Scanning electron microscopy (SEM) and electron probe microanalysis (EPMA) of the experimental new endodontic cement (NEC), described as a precursor of CEM cement, identified CaO (51.8%), SO_3_ (9.5%), P_2_O_5_ (8.5%), and SiO_2_ (6.3%) as the principal oxide constituents, together with minor inorganic components [18,19,49]. These proportions refer to the investigated experimental formulation and should not be assumed to characterize all commercially available CEM products. X-ray diffraction (XRD) analysis has additionally identified tricalcium silicate (Ca_3_SiO_5_), dicalcium silicate (Ca_2_SiO_4_), silicon oxide (SiO_2_), and zirconium oxide (ZrO_2_) [18].

One of the most distinctive compositional features of CEM cement, relative to MTA, is the presence of phosphorus as a major constituent [10,19]. In MTA and Portland cement, phosphorus is typically detected only at trace or near-threshold levels [19]. Given that hydroxyapatite is the principal inorganic component of dentin, the phosphate-rich composition of CEM cement may favor interfacial mineral similarity and thereby support cementogenesis and biomineralization [19].

Following mixing with a water-based liquid, such as distilled deionized water or phosphate-buffered saline (PBS), CEM cement undergoes a hydration reaction involving tricalcium silicate and dicalcium silicate, which leads to formation of calcium silicate hydrate gel and calcium hydroxide [10,18]. During this process, Ca^2+^, OH^−^, and phosphate ions are released, generating a calcium- and phosphate-rich environment conducive to hard tissue formation [10]. Several physicochemical properties of CEM cement have been evaluated using ISO 6876-based laboratory methods [18]. These results provide standardized reference data but should be interpreted in relation to the specific intended clinical application.

## 4. Comparative Performance of Calcium Silicate-Based Cements

### 4.1. Setting Characteristics and Handling

Setting characteristics depend on the specific commercial formulation, experimental protocol, and definition of the setting endpoint. In a direct laboratory comparison, Biodentine reached final setting earlier than ProRoot MTA [50]. However, reported setting times vary considerably among MTA Angelus, White MTA Angelus, and different ERRM formulations [14,15,16,30,31,51,52,53]. Consequently, these findings do not support a universal ranking of the four material groups according to setting time.

The prolonged setting time of conventional ProRoot MTA is considered one of its main clinical limitations because it increases the period during which the material remains susceptible to environmental contamination and washout. However, final setting time differs substantially among MTA formulations and experimental protocols. In the studies summarized in Table 1, reported final setting times for ProRoot MTA ranged from 228.33 to 329 min, with a value of 228.33 ± 2.88 min reported in one ISO-based protocol [50]. In contrast, MTA Angelus demonstrated a markedly shorter final setting time of 18.0 ± 1.0 min [51], whereas White MTA Angelus showed values of 209 ± 1.58 and 236.12 ± 0.58 min in two other experimental models [52,53]. These findings emphasize that prolonged setting should not be regarded as a uniform characteristic of all MTA formulations.

Biodentine provides a substantially faster setting profile than conventional ProRoot MTA. Experimental final-setting values of 35.4 ± 5.55, 62.42 ± 0.72, and 85.66 ± 6.03 min have been reported under different protocols [50,52,53]. Manufacturer-declared handling times and initial-setting measurements should be distinguished from final setting. Despite these inter-study discrepancies, Biodentine generally exhibits a relatively short setting time, which may reduce the risk of material displacement or washout and improve early marginal adaptation, thereby facilitating formation of an initial biological seal against bacterial ingress.

Setting and working times reported for CEM-related materials are formulation- and protocol-specific. The experimental new endodontic cement (NEC), a precursor of CEM [18], had a reported setting time of 50 ± 7.5 min and a working time of 4.5 ± 0.77 min [49]; a hand-mixed CEM preparation showed a setting time of 52.83 ± 0.60 min in an independent study [54]. Importantly, the Methods of [49] describe three measurements per material, whereas its Table 2 lists n = 5 for NEC and n = 3 for MTA. This unresolved discrepancy limits interpretation of the reported precision. Hydration and subsequent mechanical maturation depend on moisture conditions [19,55]. Neither the NEC measurements nor their study-specific uncertainty should be generalized to all commercial CEM products.

ERRM is a moisture-activated hydraulic material whose setting characteristics depend on the specific formulation, environmental humidity, and experimental conditions [10,14,15,16]. Previous reports have described setting times of approximately 2–4 h, with substantially prolonged setting under specific high-humidity conditions, although these values should not be generalized to all ERRM formulations [10]. According to the manufacturer’s instructions, the minimum setting time under normal conditions is 2 h for conventional BC RRM formulations [14,15] and 10 min for BC RRM Fast-Set Putty [16]. However, laboratory investigations have reported different final setting times, including 208 ± 10 min for conventional ERRM Putty [31] and 514 ± 79 min for the product designated ERRM Fast by Lee et al. [30]. These differences reflect formulation-specific characteristics and variations in experimental protocols rather than directly comparable setting performance. The premixed presentation and handling characteristics of ERRM have also been discussed in clinical and laboratory studies [45,56]. Nevertheless, working time should not be generalized across injectable, conventional putty, and fast-setting formulations. Therefore, manufacturer-reported setting times, experimentally determined final setting times, and working times should be distinguished and interpreted according to the specific product and testing conditions.

Setting characteristics may also be altered through experimental modifications. In an in vitro study, the addition of 5% CaCl_2_ or CaO shortened the final setting time of MTA Angelus, whereas 5% calcium nitrate reduced the initial setting time of CEM cement (BioniqueDent) [57]. These findings concern experimentally modified formulations and should not be extrapolated to unmodified commercial products or interpreted as evidence of improved clinical outcomes.

Clinically, formulation-dependent setting times affect the risk of early washout and the timing of subsequent restorative procedures. Premixed ERRM simplifies delivery but requires adequate moisture for hydration. Regardless of formulation, the cement should be protected during initial maturation; faster laboratory setting is not, on its own, evidence of superior clinical outcomes.

### 4.2. Physicochemical Properties

ISO 6876:2025 defines two categories of endodontic sealing materials: Type 1, intended for conventional orthograde root canal sealing, and Type 2, intended for other procedures, including apexification, perforation repair, resorption treatment, and retrograde root-end filling [58]. Although Type 2 materials may also be used in vital pulp therapy, the standard does not establish requirements specific to this indication. For both types, the standard specifies a maximum solubility and disintegration of 3.0% by mass and a minimum radiopacity equivalent to 3.0 mm Al for 1 mm thick specimens when tested using its specified methods. Flow (minimum 17 mm) and film-thickness (maximum 50 µm) requirements apply only to Type 1 materials. Since the studies included in this review used different protocols, including earlier versions of ISO 6876, their results are interpreted as study-specific laboratory findings rather than as direct assessments of compliance with ISO 6876:2025. Furthermore, laboratory thresholds should not be considered standalone indicators of clinical suitability.

#### 4.2.1. Sealing Ability and Microleakage

Sealing performance differs among calcium silicate-based cements as a function of porosity, particle size, interfacial mineralization, dimensional stability, and environmental conditions. Although all four materials demonstrate clinically relevant sealing potential, the mechanisms underlying this performance are not identical.

Reported marginal adaptation values for MTA-based materials vary according to formulation and experimental model. ProRoot MTA demonstrated a marginal gap of 1.17 ± 2.03 µm in one study based on SEM examination of one tooth per material [59], whereas NeoMTA showed values of 6.98 ± 2.80 µm at 1 mm and 4.85 ± 1.40 µm at 3 mm in a different model [60]. These values should therefore not be combined into a single numerical range for ProRoot MTA. ProRoot MTA has also demonstrated an apparent porosity of 29.36 ± 0.78% [41], whereas a separate micro-CT study reported a porosity of 4.95 ± 0.12% for White ProRoot MTA [61], reflecting substantial differences in porosity definitions and measurement methods. Mean microleakage values reported for MTA-based materials vary considerably among studies [62]; nevertheless, microleakage is generally low, supporting an adequate marginal seal. Interestingly, this parameter may change over time, with an initial increase followed by a subsequent decrease during longer observation periods, which may reflect progressive maturation of the material and interfacial mineral deposition. In a separate laboratory perforation study, EndoSeal MTA, a root canal sealer, was compared with ProRoot MTA, a repair cement, for bacterial microleakage [63]. This comparison involves different product categories and should not be interpreted as evidence that MTA-based sealers and repair cements have interchangeable properties or clinical indications.

Biodentine also demonstrates satisfactory marginal adaptation, although its interfacial characteristics differ from those of MTA. Biodentine exhibits an apparent porosity of approximately 22.93% [41] and relatively low structural porosity of approximately 4.71% [61]. This porosity may facilitate diffusion of calcium ions, which subsequently interact with phosphate ions to form hydroxyapatite-like deposits, thereby contributing to the bioactivity of the material while maintaining sufficient mechanical integrity. Reported marginal adaptation values for Biodentine are likewise method-dependent. A value of 16.9 ± 6.13 µm was reported in one experimental model based on SEM examination of one tooth per material [59], whereas values of 6.33 ± 1.30 µm at 1 mm and 4.62 ± 1.47 µm at 3 mm were obtained in a simulated immature-tooth model [60]. In a longitudinal leakage study, mean fluid-filtration leakage increased from 0.20 ± 0.42 µL/min after 24 h to 2.10 ± 0.57 µL/min after 1 month (mean ± SD; n = 10 teeth per material) [62]. Additional spectrophotometric leakage values after 72 h are summarized separately in Table 1 [64]. These differences illustrate the strong influence of cavity geometry, measurement method, and observation time on sealing-related outcomes.

The sealing behavior of selected premixed ERRM formulations has been associated with moisture-dependent hydration, penetration into dentinal tubules, and formation of apatite-like interfacial deposits under laboratory conditions [10]. However, these processes have not been demonstrated equivalently across injectable, conventional putty, and fast-setting formulations. Reports of sealing performance comparable to MTA originate from specific in vitro models and should be interpreted in relation to the tested products and experimental conditions rather than generalized to the entire ERRM family [10].

CEM cement likewise demonstrates favorable adaptation to root canal walls, although its sealing behavior appears to be strongly influenced by particle size, flowability, film thickness, and hydration-driven mineral deposition. Its smaller particle size, compared with MTA, contributes to improved flowability, reduced film thickness, and potentially enhanced adaptation to cavity walls [18,19]. A substantial proportion of particles ranges from 0.5 to 2.5 μm, whereas the internal diameter of dentinal tubules is approximately 2–5 μm, allowing penetration into dentinal microstructures [19]. Mild setting expansion may further enhance adaptation to cavity walls [19]. In addition, release of Ca^2+^, phosphate, and OH^−^ ions promotes hydroxyapatite formation at the material–dentin interface, thereby contributing to development of an additional biological seal [19,25,65].

Environmental contamination further differentiates the sealing behavior of these materials. CEM cement has demonstrated a significantly better seal than MTA in both dry and saliva-contaminated environments [48], whereas under blood-contaminated conditions its sealing ability appears broadly comparable to that of MTA [19,66]. Comparative evaluations using methylene blue dye and Enterococcus faecalis leakage models have likewise shown that the apical sealing capacity of CEM cement is similar to that of white MTA, gray MTA, and Portland cement [19,65]. Although blood contamination increases microleakage, CEM cement appears to maintain relatively stable sealing performance in saliva-rich environments, potentially because phosphate availability promotes hydroxyapatite crystal formation [10,48].

#### 4.2.2. Solubility and Dimensional Stability

Solubility is a critical determinant of long-term material stability because calcium silicate-based cements remain in direct contact with periapical and tissue fluids. Excessive dissolution may compromise sealing integrity, although a certain degree of ionic release is also fundamental to bioactivity.

Reported 24 h solubility values for ProRoot MTA range from approximately 0.82% to 10.89% across the studies summarized in Table 1. In studies applying ISO 6876-based laboratory methods, properly matured ProRoot MTA frequently demonstrated relatively low solubility, whereas premature exposure to aqueous media could markedly increase apparent mass loss [41,50]. The 3% value used in several cited studies represents the acceptance threshold of the ISO protocol applied in those experiments; values exceeding this benchmark indicate greater laboratory solubility but should not, in isolation, be interpreted as evidence of clinical inadequacy in repair or pulp-related applications.

Biodentine demonstrates a highly protocol-dependent solubility profile. Reported 24 h values range from approximately 1.46% to 11.83% across the studies summarized in Table 1. In the ISO-based protocol used by Kaup et al., solubility was 2.647 ± 0.583%, remaining below the 3% benchmark applied in that study [50]. At longer observation periods, values of 4.610 ± 1.402% and 7.34 ± 2.08% have been reported after 28 and 30 days of immersion, respectively [50,67]. This broad variability indicates that apparent Biodentine solubility is strongly dependent on specimen preparation, maturation before immersion, and test methodology and should not be interpreted as a single intrinsic material value.

For conventional CEM cement used as the control formulation without zinc oxide nanoparticles, Nabavizadeh et al. [68] reported solubility values of 0.0186 ± 0.006% and 0.0124 ± 0.008% (mean ± SD; n = 10) after 2 and 7 days of specimen maturation, respectively. In both cases, solubility was determined following a separate 15 h immersion in deionized water at 37 °C. These values therefore reflect different maturation conditions rather than different immersion durations and should not be generalized to other CEM formulations or testing protocols. In a separate investigation, the experimental NEC formulation exhibited a mean dimensional change of 0.075 ± 0.032 mm after setting [18,49]. This precursor-specific result should not be generalized to commercial CEM cements and is subject to the unresolved sample-count discrepancy in [49] (see Section 4.1).

Directly comparable long-term solubility data for ERRM formulations remain limited, particularly across standardized maturation and immersion protocols.

#### 4.2.3. pH and Ion Release

All four materials generate an alkaline environment during hydration, but they differ substantially in the magnitude, kinetics, and composition of ion release. These differences are biologically important because they influence antimicrobial activity, interfacial mineralization, and cellular responses.

Alkalization by MTA is strongly dependent on formulation and experimental conditions. At 3 h, reported pH values for ProRoot MTA ranged from 9.93 [69] to 10.94 ± 0.40 [70], whereas White MTA Angelus showed a value of 9.64 ± 0.24 [52]. A pH of approximately 12.5 for the original MTA formulation is summarized in Table 1. After 7 days, ProRoot MTA values remained heterogeneous, ranging from 7.39 to 11.97 as summarized in Table 1, while White MTA Angelus showed a pH of 10.41 ± 0.31 [52]. Thus, the temporal alkalization profile cannot be represented by a single range applicable to all MTA formulations.

Reported pH values require careful interpretation of the medium and sampling time. Herrera-Trinidad et al. [71] listed values of 7.41 ± 0.02 for ProRoot MTA and 7.48 ± 0.12 for Biodentine under a 3 h heading in their Results, but the Methods specify 24 h as the first measurement. The timing of these two observations is therefore unresolved and they should not be treated as confirmed 3 h data. By comparison, Al-Sherbiny et al. [70] reported pH values of 10.94 ± 0.40 and 11.68 ± 0.40 after 3 h in deionized water. Differences in buffering medium, measurement schedule, and methodology preclude direct attribution of the observed disparity to the cements alone.

Calcium ion release shows similar inter-study variability. At 3 h, ProRoot MTA released approximately 26.9–27.4 ppm of Ca^2+^ [41,70]. After 7 days, reported ProRoot MTA values ranged from 17.2 ± 6.8 to 33.10 ± 11.25 ppm [41,42], whereas a higher value of 53.47 ± 8.81 mg/L was reported for CPM MTA mixed with distilled water [72]. The latter value should therefore not be interpreted as a ProRoot MTA result.

Compared with ProRoot MTA, Biodentine generally demonstrates pronounced early calcium release, although both pH and ion-release values vary substantially among studies. At 3 h, reported pH values ranged from 9.41 [52] to 11.68 [70], while 7-day values ranged from 7.93 to 11.11 (Table 1). The lower pH value of 7.48 reported in [71] was excluded from the 3 h range because its sampling time remains uncertain. Calcium ion concentrations of approximately 95 ppm have been reported after 3 h [41,70]. After 7 days, reported values range from approximately 43.2 ppm [41,70] to 97.74 ± 16.85 ppm [42]. These findings support a pronounced ionic response but also demonstrate that its magnitude is strongly influenced by experimental conditions.

ERRM exhibits a formulation-dependent alkaline response. In a comparative in vitro investigation, the fast-setting formulation (ESF) demonstrated a higher pH than regular-setting ERRM (ES) during the seven-day observation period [17]. Therefore, pH values should be interpreted according to the tested formulation, measurement medium, and observation time rather than treated as a fixed characteristic of all ERRM products.

In the same study, regular-setting ES exhibited a higher calcium concentration and more homogeneous surface distribution in the fresh material, whereas ESF demonstrated significantly greater Ca^2+^ release over seven days [17]. Following immersion in phosphate-buffered saline, ESF also exhibited a higher Ca/P ratio and more pronounced hydroxyapatite crystal formation than ES. These findings highlight formulation-dependent differences in physicochemical bioactivity, although their implications for clinical performance require further investigation.

CEM cement differs from the other materials by releasing not only calcium and hydroxyl ions but also substantial amounts of phosphate. The alkalization profile of CEM cement also depends on specimen maturation and measurement conditions. In conventional CEM control specimens (n = 10; values expressed as mean ± SD) evaluated after 2 days of maturation at 37 °C and 100% humidity, pH values of 11.31 ± 0.26 after 5 h of immersion and 9.75 ± 0.32 after 24 h were reported. When specimens were evaluated after 7 days of maturation, the corresponding values were 11.34 ± 0.10 and 9.67 ± 0.35 [68]. These data demonstrate persistent alkalizing activity but should not be interpreted as a simple temporal decline from 5 h to 7 days because maturation duration and immersion time represent separate experimental variables. During hydration, calcium silicates, calcium phosphate, and calcium oxide contribute to the generation of calcium hydroxide and subsequent release of Ca^2+^, phosphate, and OH^−^ ions [19]. Phosphate release is particularly noteworthy, as CEM cement has been shown to exhibit higher early phosphate ion release than several other bioceramic materials during the first hour after mixing [18]. This ion-rich environment promotes hydroxyapatite formation even in normal saline [19]. A separate laboratory study evaluated calcium release from CEM mixed with its proprietary liquid or experimental propylene glycol mixtures [73].

#### 4.2.4. Radiopacity

Radiopacity differs substantially among calcium silicate-based cements and is determined largely by the type and concentration of radiopacifying agents incorporated into their formulations.

In MTA, radiopacity is primarily provided by bismuth oxide. Reported values for ProRoot MTA range from 4.32 to 7.52 mm Al [69,74]. These values are above the 3 mm Al benchmark commonly applied in ISO 6876-based radiopacity testing and therefore indicate favorable radiographic detectability under the respective experimental conditions.

Biodentine uses zirconium oxide rather than bismuth oxide as a radiopacifier. Reported radiopacity values for Biodentine vary considerably, ranging from approximately 1.50 to 4.1 mm Al across the studies summarized in Table 1 [40,50,74]. Several studies reported values below the 3 mm Al benchmark, whereas other experimental conditions yielded values above this level. These differences further demonstrate the influence of specimen thickness and radiographic methodology and should not be interpreted as a single fixed radiopacity value.

ERRM Fast demonstrated a radiopacity of 5.5 ± 0.8 mm Al [30], attributable to zirconium oxide and tantalum-containing radiopacifiers. In the same comparative study, this value exceeded Biodentine and showed no statistically significant difference from ProRoot MTA.

CEM cement demonstrates radiopacity greater than that of natural dental tissues, with a reported value of approximately 2.227 mm Al [18]. The reported radiopacity of CEM cement may fall below the 3 mm Al benchmark used in standardized laboratory testing [18]. This should be interpreted as a comparative physicochemical characteristic rather than as a standalone criterion of clinical suitability, particularly because measured radiopacity is influenced by imaging modality and scanner-specific calibration.

Radiopacity should be interpreted at the level of the tested formulation rather than the broader material family. In one direct laboratory comparison, ProRoot MTA and the formulation designated ERRM Fast demonstrated higher radiopacity than Biodentine under the experimental conditions used [30]. These findings cannot be generalized to other MTA or ERRM formulations, particularly because radiopacifier composition and imaging protocols vary. Bismuth oxide-containing formulations may provide high radiopacity but are associated with discoloration and potential biological concerns [75,76], whereas zirconium- and tantalum-containing alternatives present different physicochemical trade-offs [76].

The principal physicochemical parameters reported for the four materials are summarized in Table 1, with source-specific sample sizes, testing conditions, and statistical definitions in Supplementary Table S1.

**Table 1:** Reported physicochemical properties of MTA formulations, Biodentine, CEM cement, and ERRM formulations. The table includes measurements from direct within-study comparisons and independent investigations. Within-study comparisons are interpretable only when the same endpoint was evaluated under comparable experimental conditions. Values obtained from different studies are presented descriptively and should not be used for direct numerical ranking.

Property	Time Point/Measurement	MTA Formulations	Biodentine	CEM Cement	ERRM Formulations
Initial setting time (min)	Study-specific method	MTA Angelus: 10.3 ± 1.5 [51]	30.00 ± 0.00 [77]	Conventional CEM (without ZON): 11.0 ± 0.0 [68]	ERRM Putty: 61.8 ± 2.5 [31]
Final setting time (min)	Endpoint as defined by each study	MTA Angelus: 18.0 ± 1.0 [51]; White MTA Angelus: 209 ± 1.58 [52], 236.12 ± 0.58 [53]; ProRoot MTA: 228.33 ± 2.88 [50], 329 ± 31 [30]	18 ± 8 [30]; 35.4 ± 5.55 [52]; 45 [40]; 62.42 ± 0.72 [53]; 85.66 ± 6.03 [50]	Conventional CEM (without ZON): 45.0 ± 6.64 [68]	ERRM Putty: 208 ± 10 [31]; ERRM Fast: 514 ± 79 [30]
Setting time (min)	Initial/final endpoint not differentiated	—	—	Experimental CEM precursor (NEC): 50 ± 7.5 [49] ‡; hand-mixed CEM: 52.83 ± 0.60 [54]	—
Solubility (%)	24 h	ProRoot MTA: 0.880 ± 0.237 [50]; 0.82 ± 0.34 [78]; 1.51 [79]; 2.27 ± 0.0 [80]; 3.27 ± 0.85 [70]; 10.89 ± 0.48 [41]	1.46 [79]; 2.62 ± 0.25 [81]; 2.647 ± 0.583 [50]; 2.94 ± 0.09 [70]; 11.83 ± 0.52 [41]	—	EndoSequence Root Repair Material: 1.39 [79]
Solubility (%)	15 h immersion after 2 or 7 d of maturation	—	—	Conventional CEM (without ZON): 2 d, 0.0186 ± 0.006; 7 d, 0.0124 ± 0.008 [68]	—
	7 d	MTA Angelus: 1.757 [82]	—	—	—
	28 d or 30 d immersion, as specified	ProRoot MTA: 28 d, 1.144 ± 0.328 [50], 3.51 ± 0.03 [80]; MTA Angelus: 30 d, 3.471 [82]	28 d, 4.610 ± 1.402 [50]; 30 d, 7.34 ± 2.08 [67]	—	—
pH	3 h (see † for [71])	ProRoot MTA: 7.41 ± 0.02 [71] †; 9.93 [69]; 10.94 ± 0.4 [70]; White MTA Angelus: 9.64 ± 0.24 [52]; original MTA formulation: 12.5 [83]	7.48 ± 0.12 [71] †; 9.41 ± 0.65 [52]; 9.85 ± 0.11 [84]; 11.68 ± 0.4 [70]	CEM + 100% CEM liquid (control): 10.13 ± 0.89 [73]	—
	5 h immersion after 2 or 7 d maturation	—	—	Conventional CEM (without ZON): 11.31 ± 0.26 after 2 d maturation; 11.34 ± 0.10 after 7 d maturation [68]	—
	24 h immersion; maturation as specified	—	—	CEM + 100% CEM liquid (control): 8.70 ± 0.45 [73]; conventional CEM (without ZON): 9.75 ± 0.32 after 2 d maturation; 9.67 ± 0.35 after 7 d maturation [68]	—
	72 h	—	—	CEM + 100% CEM liquid (control): 7.35 ± 0.08 [73]	—
	7 d	ProRoot MTA: 7.39 ± 0.07 [71]; 8 [69]; 8.64 ± 0.4 [70]; 11.97 ± 0.06 [78]; White MTA Angelus: 10.41 ± 0.31 [52]	7.93 ± 0.02 [71]; 9.238 ± 0.3 [70]; 10.69 ± 0.18 [52]; 11.11 ± 0.48 [84]	—	—
Radiopacity (mm Al)	Study-specific methods	ProRoot MTA: 4.32 ± 0.17 [74]; 4.8 ± 1.0 [30]; 6.40 ± 0.06 [50]; 7.52 ± 0.15 [69]; White MTA Angelus: 4.93 ± 0.94 [52]	1.50 ± 0.10 [50]; 1.5 ± 0.2 [30]; 2.29 ± 0.21 [74]; 2.79 ± 0.27 [52]; 2.82 ± 0.11 [81]; 4.1 at 1 d and 3.3 at 28 d in HBSS [40]	CEM: 2.227 (secondary report) [18]	ERRM Fast: 5.5 ± 0.8 [30]
Ca^2+^ release (ppm or mg/L, as reported)	3 h	ProRoot MTA: 26.9 ± 3.4 ppm [70]; 27.4 ± 5.4 ppm [41]	95.3 ± 13.0 ppm [41]; 95.68 ± 7.5 ppm [70]	CEM + 100% CEM liquid (control): 10.44 ± 3.1 mg/L [73]	—
	7 d	ProRoot MTA: 17.2 ± 6.8 ppm [41]; 18.7 ± 3.0 ppm [70]; 33.10 ± 11.25 ppm [42]; CPM MTA + distilled water: 53.47 ± 8.81 mg/L [72]	43.2 ± 2.5 ppm [70]; 43.2 ± 2.8 ppm [41]; 97.74 ± 16.85 ppm [42]	CEM + 100% CEM liquid (control): 8.94 ± 0.97 mg/L [73]	—
Microleakage–fluid filtration (µL/min)	24 h after setting	Gray ProRoot MTA: 0.80 ± 0.63 [62]	0.20 ± 0.42 [62]	—	—
	1 week after setting	Gray ProRoot MTA: 1.40 ± 0.52 [62]	1.40 ± 0.52 [62]	—	—
	1 month after setting	Gray ProRoot MTA: 0.60 ± 0.52 [62]	2.10 ± 0.57 [62]	—	—
Microleakage–linear dye penetration (mm)	1 week dye exposure	MTA, non-etched root-end cavities: 0.17 ± 0.14 [85]; MTA Angelus, intraorifice barrier: 0.238 ± 0.159 [86]	0.197 ± 0.090 [86]	Conventional CEM: 0.175 ± 0.068 [86]	—
Microleakage–spectrophotometric measurement (Abs)	72 h	MTA Angelus: carbide bur, 0.278 ± 0.042; diamond bur, 0.254 ± 0.055 [64]	Carbide bur, 0.139 ± 0.033; diamond bur, 0.153 ± 0.038 [64]	—	—
Microleakage–spectrophotometric assay (unit not reported)	Test duration not reported	Unmodified MTA: 0.8333 ± 0.21882; MTA with chitosan nanohydrogel: 0.7147 ± 0.20842 [87]	—	—	—
Apparent porosity (%)	24 h water immersion; Archimedes method	ProRoot MTA: 29.36 ± 0.78 [41]	22.93 ± 0.24 [41]	—	—
Total porosity (%)–micro-CT	24 h maturation [61,67]; 72 h [88]	White ProRoot MTA: 4.95 ± 0.12 [61]; MTA Angelus: 10.79 ± 1.02 [88]	4.71 ± 0.14 [61]; 6.36 ± 1.03 [67]; 13.04 ± 2.32 [88]	—	EndoSequence Root Repair Material: 6.11 ± 0.96 [88]
Open porosity (%)–Archimedes method	7 d in water	—	—	—	ERRM Putty: 16.7 ± 2.8 [31]
	28 d in water	—	—	—	ERRM Putty: 14.3 ± 1.1 [31]
Marginal gap (µm)	Study-specific methods	ProRoot MTA: 1.17 ± 2.03 [59]; NeoMTA: 6.98 ± 2.80 at 1 mm and 4.85 ± 1.40 at 3 mm [60]	16.9 ± 6.13 [59]; 6.33 ± 1.30 at 1 mm and 4.62 ± 1.47 at 3 mm [60]	—	—
Film thickness (mm)	Study-specific methods	ProRoot MTA: 0.58 ± 0.05 [78]; 0.452 ± 0.063 [49] ‡	—	Experimental CEM precursor (NEC): 0.174 ± 0.025 [49] ‡; hand-mixed CEM: 1.3 ± 0.05 [54]	—

For the CEM solubility data in [68], the unmodified control was CEM cement (BioniqueDent, Tehran, Iran), without zinc oxide nanoparticles. Specimens (n = 10; 7 mm diameter × 1 mm thickness) matured at 37 °C and 100% humidity for 2 or 7 days, were desiccated for 1 h before initial weighing, immersed in deionized water at 37 °C for 15 h, and desiccated again for 2 days before final weighing. Values are mean ± standard deviation (SD) of percentage loss of initial dry mass. The 2- and 7-day intervals denote maturation, not immersion duration. Methodological note. Numerical findings obtained in different studies are descriptive, not directly rankable. Supplementary Table S1 identifies source-specific formulations, specimen counts, conditioning, methods, reported variability, and missing information. The ± symbol denotes SD only where verified; otherwise its meaning is not assumed. Initial, final, and unspecified setting endpoints and manufacturer-declared handling times are not interchangeable; the secondary setting time in [68] follows that study’s definition of final setting. † The pH values labelled 3 h in the Results of [71] conflict with the Methods, which give 24 h as the first measurement; their actual timing remains uncertain. ‡ In [49], the Methods state three measurements per material, whereas Table 2 lists n = 5 for NEC and n = 3 for MTA. The sample-count discrepancy and incomplete reporting limit interpretation of the corresponding setting-time, flow, and film-thickness results. Unspecified sample sizes, dispersion measures, measurement conditions, or endpoints must not be inferred.

### 4.3. Mechanical Properties

Mechanical performance of calcium silicate-based cements develops progressively during hydration and is strongly influenced by material composition, porosity, environmental conditions, and maturation time. The four materials considered in this review differ particularly in the rate at which mechanical stability is achieved, whereas direct comparison of absolute values remains complicated by substantial methodological heterogeneity among studies.

#### 4.3.1. Compressive Strength

Compressive strength is considered a useful indicator of hydration progress and material hardening [78]. All four materials demonstrate time-dependent changes in compressive strength, although the magnitude and kinetics of this increase differ considerably.

Compressive strength values reported for MTA formulations vary widely according to formulation, maturation time, and testing protocol. At 24 h, ProRoot MTA values of 20.15 ± 0.6 and approximately 40 MPa have been reported [10,70], whereas White MTA Angelus values ranged from 18.46 ± 1.9 to 41.45 ± 0.53 MPa [52,53]. At 7 days, ProRoot MTA values ranged from approximately 24 to 68 MPa [70,78], while White MTA Angelus reached 93.44 ± 0.93 MPa in another experimental model [53]. At 21 days, approximately 67 MPa has been reported for ProRoot MTA [10], whereas White MTA Angelus values ranged from 27.68 ± 3.56 to 136.78 ± 0.26 MPa across different studies [52,53]. These large differences indicate that absolute compressive strength values should not be generalized across MTA formulations or experimental protocols. Importantly, contact with blood and saliva does not appear to significantly compromise compressive strength, suggesting that selected MTA formulations can maintain structural integrity under contaminated experimental conditions [89].

Reported compressive strength values for Biodentine differ markedly between independent studies. Lucas et al. [52] reported values of 19.78 ± 2.55 MPa after 24 h and 37.22 ± 5.27 MPa after 21 days using cylindrical specimens measuring 6 mm in diameter and 12 mm in height, tested at a crosshead speed of 0.5 mm/min. In contrast, Ravindran and Jeevanandan [53] reported substantially higher values of 230.09 ± 0.47 MPa after 24 h, 350.42 ± 0.25 MPa after 7 days, and 413.57 ± 0.73 MPa after 21 days using smaller specimens (4 mm diameter × 6 mm height) and a loading rate of 1 mm/min. Differences in specimen geometry, preparation, conditioning, and testing procedures may contribute to this discrepancy, but their individual effects have not been established. Moreover, the sample size at each destructive testing time point is not clearly specified in [53]. Consequently, these results should be interpreted as protocol-specific observations rather than combined into a representative compressive-strength range or used to establish material superiority. For context, these values should not be directly equated with the compressive strength of natural dentin, which has been reported at approximately 275–300 MPa [90].

ERRM Putty demonstrates progressive mechanical maturation, with compressive strength increasing from 107.4 ± 31.1 MPa after 7 days to 176.6 ± 22.0 MPa after 28 days in one direct experimental study [31]. In the same study, ERRM Putty showed lower porosity than the other evaluated hydraulic bioceramic materials, which may contribute to its relatively high compressive strength. These values remain specific to the Putty formulation and the experimental conditions used.

An unusually large between-study discrepancy is evident for CEM cement. Nabavizadeh et al. [68] reported a 21-day compressive strength of 5.78 ± 1.89 MPa for conventional CEM controls (n = 6), using cylindrical specimens measuring 4 mm in diameter and 6 mm in height, initially conditioned at 37 °C and 100% humidity and subsequently maintained in PBS-moistened gauze. In contrast, Shahi et al. [91] reported 267.67 ± 21.96 MPa after 21 days for hand-mixed CEM specimens (n = 6 per mixing method and time point), using 6 × 12 mm cylinders stored in distilled water. Both studies used a crosshead speed of 0.5 mm/min. Verification against the original publications confirmed that both values are reported in MPa and correspond to 21 days of maturation. Although differences in specimen geometry, mixing procedures, storage media, and testing protocols may influence the results, these factors have not been demonstrated to explain the magnitude of the discrepancy. Accordingly, the values should be interpreted as independent, protocol-specific observations rather than combined into a representative compressive-strength range. Standardized, independently replicated comparisons are required to resolve this inconsistency.

#### 4.3.2. Tensile and Flexural Behavior

Tensile and flexural properties provide complementary information on the resistance of brittle calcium silicate cements to stresses that differ from direct compression. However, comparative evidence is substantially less complete for these parameters than for compressive strength.

Direct tensile testing is difficult in brittle materials such as hydraulic cements. Therefore, diametral tensile strength (DTS) is commonly used as an indirect measure, in which a cylindrical sample is subjected to compressive force along the diametral plane, generating tensile stress perpendicular to the applied load [92]. For commercial White MTA Angelus, a diametral tensile strength of 7.60 ± 1.99 MPa has been reported [93]. In the same study, an experimental dual-cure MTA formulation demonstrated a lower value of 4.81 ± 1.15 MPa, further illustrating the formulation dependence of this mechanical parameter. DTS is strongly influenced by environmental pH. Samples stored at acidic pH values of approximately 4.5 showed the lowest DTS, whereas samples stored at pH 8.4 exhibited the highest values. This reduction under acidic conditions may result from corrosion and decomposition of calcium hydroxide, calcium sulfoaluminate, and calcium silicate hydrate phases, leading to increased porosity and reduced structural cohesion [92]. MTA containing fine-particle bismuth oxide has also been reported to exhibit higher DTS than formulations containing medium- or coarse-particle radiopacifier [92].

Flexural strength is particularly relevant when the material is subjected to restorative procedures or mechanical loading [94]. White MTA Angelus exhibited relatively low flexural strength, reaching 10.25 ± 1.6 MPa after 96 h of setting [95]. This relatively limited early resistance suggests that freshly placed MTA-based materials should be protected from excessive mechanical loading during the initial maturation period.

Biodentine demonstrates a broadly comparable but highly variable flexural profile. Reported flexural strength values range from 9.49 ± 2.90 MPa [77] to approximately 22 MPa according to manufacturer data. The higher values have been attributed to the low water-to-cement ratio enabled by the polymeric additive in the liquid component, which contributes to formation of a denser matrix with reduced porosity [40]. These findings indicate that the flexural properties of Biodentine are strongly influenced by formulation details and testing methodology.

CEM cement has also been evaluated with respect to flexural behavior. When assessed according to ISO 4049 criteria, its flexural properties differ from those of Angelus MTA and BioAggregate [18]. In the short term, exposure of root dentin to both CEM cement and ProRoot MTA has been associated with reduced dentin flexural strength in a manner broadly similar to calcium hydroxide [18]. However, a potentially relevant long-term distinction has been observed: whereas dentin exposed to calcium hydroxide and Angelus MTA demonstrated a decline in flexural strength after 1 year, samples exposed to CEM cement retained their initial strength [18]. This finding may indicate a more favorable long-term interaction with the dentin substrate.

Comparable tensile and flexural data for ERRM remain limited. Therefore, the available evidence does not support a robust ranking of all four materials according to tensile or flexural performance. More standardized head-to-head studies are required before these parameters can be reliably translated into material-selection criteria.

#### 4.3.3. Push-Out Bond Strength

Push-out bond strength to dentin is an important mechanical parameter because it reflects resistance to displacement and preservation of interfacial integrity under clinical conditions [96]. Substantial differences have been reported among calcium silicate-based cements, although adhesion is strongly dependent on maturation time, environmental pH, blood contamination, dentin preparation, and experimental protocol. Bond-strength data should also be distinguished by test configuration, because shear-bond studies involving restorative materials address a different interface from push-out dislodgement tests [97].

White ProRoot MTA exhibits relatively low early push-out bond strength, with a value of 5.34 ± 2.37 MPa reported after 24 h of contact with blood [98]. After 72 h, however, bond strength increases to approximately 9 MPa [98] and up to 11.99 ± 3.99 MPa in other studies [99]. This improvement is consistent with ongoing hydration, formation of calcium silicate hydrate and calcium hydroxide, and subsequent hydroxyapatite deposition, which enhances interfacial bonding to dentin [59,98,99]. Acidic pH significantly impairs the push-out bond strength of MTA [100], whereas CaCl_2_-accelerated formulations may demonstrate improved adhesive performance [10].

Biodentine generally achieves higher push-out bond strength at earlier time points than conventional MTA. Values of approximately 34.5 MPa after 24 h and 37.03 ± 16.16 MPa after 72 h have been reported [98]. These findings are consistent with relatively rapid material maturation and formation of a stable micromechanical interface with dentinal walls. However, the large variability among reported values indicates that the magnitude of this apparent advantage depends strongly on the experimental protocol.

In a specific 34-day phosphate-buffered saline model, the evaluated ERRM Putty formulation demonstrated a push-out bond strength of 13.54 ± 5.56 MPa [100]. Other experimental observations summarized in [10] involved different products, comparators, and test configurations, including comparisons with endodontic sealers. Accordingly, these findings support formulation- and model-specific resistance to dislodgement but do not establish a consistent mechanical advantage for all ERRM formulations.

The available data for CEM cement are more heterogeneous. Some studies have reported bond strength comparable to MTA, particularly in ultrasonically prepared root-end cavities, whereas others have documented lower values [18]. In one experimental dataset, MTA exhibited a push-out bond strength of 5.94 ± 3.99 MPa compared with 1.68 ± 0.9 MPa for CEM cement [101]. However, smear layer removal, cavity preparation technique, dentin conditioning, and formulation modifications substantially affect this parameter. Dentin conditioning with diode or Er:YAG laser irradiation has been reported to increase bond strength, while prior calcium hydroxide placement and addition of calcium chloride may also enhance adhesion over time [18].

#### 4.3.4. Microhardness

Microhardness provides an indirect measure of surface maturation and structural development of calcium silicate cements. Similar to compressive and bond strength, it changes considerably during hydration and is sensitive to environmental pH and moisture conditions.

The microhardness of ProRoot MTA increases during early maturation, although reported values differ among studies. After 24 h, values range from 26.93 ± 4.66 to approximately 37.5 HV [10,50]. After 7 days, reported values range from 53.5 ± 4.3 to 72.61 ± 1.38 HV [30,102]. These findings support progressive surface maturation but also demonstrate substantial protocol-dependent variability.

Biodentine also demonstrates substantial variation in reported microhardness. Values after 24 h range from approximately 45.4 to 62.35 HV [10,50], whereas 7-day values of 69.00 ± 0.71 and 101.2 ± 5.0 HV have been reported in different studies [30,102]. At 28 days, reported values remain heterogeneous, ranging from approximately 48.4 to 74.4 HV [40,103]. These data do not support a single stabilization range and instead reinforce the strong influence of experimental methodology on measured surface hardness.

ERRM Putty shows a slower early surface-hardness development pattern. In one study, it had the lowest initial microhardness among the evaluated bioceramic cements, which may be related to its slower early setting reaction [31]. Hardening was minimal during the first 24 h, but microhardness subsequently increased and reached values similar to those of other bioceramic materials by approximately day 4 [31]. In an in vitro study, the ERRM Putty formulation exhibited reduced microhardness following exposure to acidic conditions (butyric acid, pH 5.5) [104]. This finding should be interpreted within the tested experimental protocol rather than generalized to all ERRM formulations.

The microhardness of CEM cement likewise increases progressively with maturation and is strongly influenced by environmental conditions. Studies have demonstrated a consistent rise in surface hardness from day 1 to day 7 [18]. Because CEM requires moisture for hydration, exposure to humid conditions facilitates water uptake and promotes continued setting, thereby increasing microhardness [55]. Conversely, acidic environments negatively affect final microhardness, whereas exposure to phosphate-buffered saline has been reported to enhance it [18].

Several studies suggest relatively rapid surface-hardness development for Biodentine, while MTA, ERRM, and CEM cement show formulation- and condition-dependent maturation profiles. Importantly, acidic conditions adversely affect the microhardness of several calcium silicate-based cements, indicating that values obtained under neutral laboratory conditions may overestimate early mechanical stability in inflamed clinical environments.

The principal mechanical parameters reported for the four materials are summarized in Table 2.

**Table 2:** Reported mechanical properties of MTA formulations, Biodentine, CEM cement, and ERRM formulations. Mechanical values were obtained from independent studies using different material formulations, specimen geometries and dimensions, maturation periods, storage media, contamination conditions, loading rates, and testing protocols. Values derived from different studies should not be interpreted as direct head-to-head comparisons or used for numerical ranking. Approximate values (≈), where reported, should be interpreted cautiously. Compressive-strength results are grouped by source study; time points within a row apply only to that study. Values taken from reviews [10,105] are identified as secondary-source data.

Property/Source Study	Time Point/Condition or Protocol	MTA Formulations	Biodentine	CEM Cement	ERRM Formulations
Push-out bond strength (MPa)	24 h, blood-exposed specimens	White ProRoot MTA: 5.34 ± 2.37 [98]	≈34.5 [98]	Conventional CEM: ≈7 [98]	ERRM Putty: ≈8.4 [98]
	72 h, blood-exposed specimens	White ProRoot MTA: ≈9 [98]	37.03 ± 16.16 [98]	Conventional CEM: ≈8 [98]	ERRM Putty: ≈16 [98]
	72 h, study-specific conditions	ProRoot MTA: 11.99 ± 3.99 [99]	14.94 ± 5.94 [99]	Hand-mixed CEM: 4.86 ± 1.41 [105] (secondary)	—
	72 h, root-end model	MTA: 5.94 ± 3.99 [101]	—	CEM cement: 1.68 ± 0.9 [101]	—
	34 d, phosphate-buffered saline	—	—	—	ERRM Putty: 13.54 ± 5.56 [100]
Compressive strength (MPa) Lucas et al. [52]	24 h; 21 d 6 × 12 mm; 37 °C, 95% RH; 0.5 mm/min	White MTA Angelus: 24 h: 18.46 ± 1.9 21 d: 27.68 ± 3.56 [52]	24 h: 19.78 ± 2.55 21 d: 37.22 ± 5.27 [52]	—	—
Ravindran and Jeevanandan [53]	24 h; 7 d; 21 d 4 × 6 mm; 37 °C; 1 mm/min; n per destructive time point unclear	White MTA Angelus: 24 h: 41.45 ± 0.53 7 d: 93.44 ± 0.93 21 d: 136.78 ± 0.26 [53]	24 h: 230.09 ± 0.47 7 d: 350.42 ± 0.25 21 d: 413.57 ± 0.73 [53]	—	—
Nabavizadeh et al. [68]	24 h; 21 d 4 × 6 mm; PBS-moistened gauze; 0.5 mm/min; n = 6	—	—	CEM control without ZON (mean ± SD): 24 h: 0.76 ± 0.35 21 d: 5.78 ± 1.89 [68]	—
Shahi et al. [91] Primary study	21 d 6 × 12 mm; distilled water; 37 °C; 0.5 mm/min; n = 6	—	—	Hand-mixed CEM: 21 d: 267.67 ± 21.96 [91]	—
Al-Sherbiny et al. [70]	24 h; 7 d	ProRoot MTA: 24 h: 20.15 ± 0.6 7 d: 24.23 ± 2.2 [70]	24 h: 54.27 ± 6.1 7 d: 60.22 ± 1.4 [70]	—	—
Guo et al. [31]	7 d; 28 d	—	—	—	ERRM Putty: 7 d: 107.4 ± 31.1 28 d: 176.6 ± 22.0 [31]
Kang et al. [78]	7 d	ProRoot MTA: 7 d: 68.21 ± 16.22 [78]	—	—	—
Agrawal et al. [81]	24 h; 21 d	—	24 h: 41.08 ± 1.84 21 d: 88.93 ± 3.39 [81]	—	—
Olcay et al. [102]	7 d	ProRoot MTA: 7 d: 24.97 ± 4.66 [102]	7 d: 59.81 ± 18.13 [102]	—	—
Grech et al. [40]	28 d	—	28 d: 67.18 [40]	—	—
Dawood et al. [10] Review (secondary)	24 h; 21 d	ProRoot MTA: 24 h: 40 21 d: 67 [10]	24 h: 61.3–78.5 [10]	—	—
Diametral tensile strength (MPa)	Study-specific conditions	White MTA Angelus: 7.60 ± 1.99 [93]; experimental dual-cure MTA: 4.81 ± 1.15 [93]	—	—	—
Flexural strength (MPa)	96 h	White MTA Angelus: 10.25 ± 1.6 [95]	—	Conventional CEM: 9.09 ± 1.16 [95]	—
	Study-specific conditions	—	9.49 ± 2.90 [77]	—	—
Microhardness	24 h	ProRoot MTA: 26.93 ± 4.66 HV [50]; 37.5 HV [10] (secondary)	62.35 ± 11.55 HV [50]; 45.4 HV [10] (secondary)	—	ERRM Putty: ≈5 HV [31]
	4 d	—	—	CEM + 100% CEM liquid (control): 31.09 ± 3.22 HV [73]	ERRM Putty: ≈44 HV [31]
	7 d	ProRoot MTA: 53.5 ± 4.3 HV [30]; 72.61 ± 1.38 HV [102]	69.00 ± 0.71 HV [102]; 101.2 ± 5.0 HV [30]	—	ERRM Putty: ≈45 HV [31]; ERRM Fast: 42.6 ± 13.7 HV [30]
	21 d; Vickers, 300 g load, 10 s [73]	—	—	CEM + 100% CEM liquid (control): 30.05 ± 0.91 HV [73]	—
	21 d; Vickers, 500 g load [106]	White MTA Angelus: 129.50 ± 3.80 HV [106]	—	Conventional CEM: 129.82 ± 3.80 HV [106]	—
	28 d	White MTA Angelus: ≈69.9 HV [103]	48.4 [40]; ≈74.4 HV [103]	—	ERRM Putty: ≈46 HV [31]

For the unmodified CEM control in [68], compressive strength is mean ± SD (n = 6 per group/time point); specimens (4 mm diameter × 6 mm height) were kept for 3 h at 37 °C/100% humidity, then matured with PBS-moistened gauze to 24 h or 21 d and tested at 0.5 mm/min. These times denote maturation, not the 15 h solubility immersion in Table 1. Compressive-strength dimensions are diameter × height; RH, relative humidity. Shahi et al. [91] report mean ± SD (n = 6 per mixing method/time point). Study rows are independent. HV denotes Vickers hardness; values in [106], originally labelled kg/mm^2^, are expressed as HV without numerical conversion. The 21 d hardness results from [73] and [106] are separated because test loads and specimen protocols differ. Some primary reports do not clearly state the independent specimen count for each destructive time point (e.g., [53]), and the statistical meaning of every reported ± value is not always specified. Missing information was not imputed; all measurements remain descriptive and are unsuitable for a universal material ranking.

### 4.4. Biological Compatibility and Tissue Response

Calcium silicate-based cements may contact pulp, periodontal, periapical, and stem cell populations. Their biological compatibility is assessed through cell viability, inflammatory signaling, genotoxicity, and formulation-specific effects.

#### 4.4.1. Cytotoxicity and Cell Viability

In vitro studies generally indicate low cytotoxicity for all four materials, although cellular responses depend on material formulation, setting status, exposure time, extract concentration, and the cell type used.

ProRoot MTA exhibits low cytotoxicity and favorable biocompatibility. In human periodontal ligament fibroblasts, exposure to MTA maintained high cell viability without toxic effects [69]. Similarly, in human alveolar bone marrow-derived stem cells, MTA preserved cell viability during a 14-day culture period and promoted osteogenic activity and mineral deposition [107]. MTA also demonstrated low cytotoxicity in peripheral blood monocyte–macrophage cells, although a slight tendency to induce apoptosis after 48 h has been reported [108]. Overall, these findings indicate a favorable cytocompatibility profile, although the translational relevance of isolated in vitro viability data should be interpreted cautiously.

Biodentine demonstrates a broadly comparable profile. No significant cytotoxicity or induction of apoptosis has been reported in peripheral blood monocyte/macrophage cells [108]. Similar findings were observed in human dental pulp stem cells (hDPSCs) exposed to Biodentine-conditioned media. Although cell viability decreased to approximately 70% under some experimental conditions, this value remained within the threshold for non-cytotoxic materials according to ISO 10993-5 [109]. Studies involving human bone marrow stem cells derived from mandibular bone have likewise confirmed the absence of significant cytotoxic effects [107], and favorable cytocompatibility has also been demonstrated in Balb/c 3T3 fibroblast-based models [110].

The available experimental evidence summarized in [10] suggests generally favorable cytocompatibility of the evaluated ERRM preparations across several fibroblast and osteoblast-like cell models. However, selected experiments involving Saos-2 cells reported lower viability or alkaline phosphatase activity than the corresponding MTA comparators [10]. Because material formulations, setting status, exposure conditions, and cellular models differed, these findings should not be interpreted as evidence of equivalent biological behavior across regular-setting, injectable, putty, and fast-setting ERRM formulations.

CEM cement is also characterized by a favorable biological profile and demonstrates biocompatibility comparable to, and in some experimental settings greater than, that of ProRoot MTA [10,18]. Studies using L929 mouse fibroblasts, human gingival fibroblasts, dental pulp stem cells, and stem cells from the apical papilla have consistently shown good cell viability following exposure to CEM cement [18,19]. Set CEM cement generally demonstrates better cellular compatibility than freshly mixed formulations, although fresh CEM remains broadly comparable to fresh MTA in terms of initial toxicity [18,19]. In some regenerative models, CEM has shown lower cytotoxicity than ProRoot MTA after 48 h of incubation [18].

Interpretation also depends on experimental conditions rather than on the material alone. In human dental pulp stem cells, exposure to MTA or Biodentine at concentrations of 0.002, 0.02, and 0.2 mg/mL produced concentration- and time-dependent differences in ALP and DSPP expression over 24–72 h [7]. In another study, human pulp cells were exposed to extraction media diluted to 10%, 25%, 50%, or 100%, with viability assessed after 24 h and proliferation monitored over seven days [109]. Differences in extract preparation, dilution, material setting status, exposure duration, and cell model limit comparisons between studies. Neither increased marker expression nor short-term cell viability establishes improved clinical pulp healing.

#### 4.4.2. Inflammatory Response

Beyond direct effects on cell viability, calcium silicate-based cements may modulate inflammatory signaling, which represents an important component of pulp healing and subsequent mineralized tissue formation.

ProRoot MTA influences inflammatory signaling, differentiation, and mineralization in human dental pulp stem cells, including modulation of S100A family gene expression. Experimental studies have demonstrated increased IL-8 mRNA expression after 24 and 72 h and IL-6 expression after 72 h following exposure to MTA-conditioned media [7]. These findings suggest a time-dependent modulation of inflammatory signaling, although their implications for pulp healing require further investigation.

Biodentine also modulates inflammatory cytokine signaling, although the direction of this response appears to depend on the experimental model. In a three-dimensional fibroblast model, Biodentine was associated with increased IL-1α production, whereas studies using LPS-challenged THP-1-derived macrophages and dental pulp stem cells demonstrated downregulation of pro-inflammatory cytokines, including IL-8, together with enhancement of an anti-inflammatory regenerative profile [110,111]. These findings suggest that Biodentine induces a context-dependent immunomodulatory response rather than a uniformly pro-inflammatory effect.

Available comparative evidence regarding inflammatory signaling induced by ERRM and CEM cement is less extensive in the studies considered here. Therefore, although both materials demonstrate favorable overall biocompatibility, direct comparison of their immunomodulatory profiles with MTA and Biodentine remains limited.

A transient inflammatory response is not necessarily adverse; its magnitude, duration, and resolution may be more informative than the presence or absence of cytokine induction.

#### 4.4.3. Genotoxicity and Material-Associated Safety Concerns

Genotoxicity data are more limited than conventional cytotoxicity data, and the available findings indicate that biological safety may depend not only on the calcium silicate matrix itself but also on additional formulation components, particularly radiopacifiers.

MTA has demonstrated no significant genotoxicity in peripheral blood monocyte–macrophage cells in some experimental models [108]. However, recent studies have raised concerns regarding bismuth oxide, the radiopacifier used in conventional MTA formulations. Bismuth oxide may leach from the MTA matrix and exhibit cytotoxic effects under specific conditions [75]. Thus, although MTA is generally regarded as biologically compatible, its safety profile may be influenced by the presence and behavior of the radiopacifying phase. These observations support continued interest in alternative radiopacifiers, particularly because bismuth oxide is additionally associated with dentin discoloration and possible systemic migration [75].

Biodentine has shown no evidence of genotoxicity in peripheral blood monocyte/macrophage cell models [108]. Its use of zirconium oxide rather than bismuth oxide may avoid some of the formulation-associated concerns identified for conventional MTA, although direct long-term comparative safety data remain limited.

In an MC3T3 osteoblastic cell model, the EndoSequence BC Root Repair formulation evaluated by Abrão et al. [112] showed greater genotoxicity than MTA Angelus and MTA Repair HP after seven days of exposure. This finding is specific to the evaluated formulation and experimental conditions and should not be extrapolated to other ERRM products.

CEM cement has demonstrated a generally favorable genotoxicity profile. Ames test data indicate that CEM cement is non-mutagenic and does not induce significant mutations [18]. Some concentration-dependent genotoxic effects have nevertheless been reported in L929 fibroblasts, with an unusual pattern in which higher concentrations were associated with lower genotoxicity than lower concentrations [18]. Such findings emphasize the importance of experimental conditions when interpreting material-associated genetic toxicity.

Current data reveal no major genotoxicity concern but remain limited. Formulation components require separate assessment because their biological effects may differ from those of the calcium silicate matrix.

### 4.5. Bioactivity and Regenerative Potential

These cements share bioactivity associated with calcium release, alkalization, and interfacial mineralization. However, phosphate availability depends on material composition and the surrounding medium, and the mechanisms and kinetics of mineralized interface formation differ between formulations.

The bioactivity of MTA-based formulations is closely associated with calcium ion release derived from calcium hydroxide dissociation [41,113]. These ions react with phosphate ions present in tissue fluids, resulting in the formation of calcium phosphate and hydroxyapatite on the material surface. This process promotes biomineralization and interfacial integration between the material and surrounding tissues, progressively filling the material–dentin interface with mineralized deposits.

Calcium release from selected MTA formulations has been linked to mineralization-related processes involving osteopontin, BMP-2, and alkaline phosphatase activity, as discussed in [41]. These mechanistic associations should be distinguished from direct evidence of tissue repair. In a short-term rat pulp-capping model, ProRoot MTA was evaluated for inflammatory responses and hard-tissue formation after 7 and 28 days [42]. Such preclinical findings provide information beyond isolated cellular responses but do not establish long-term pulp healing or clinical superiority in humans.

Biodentine shares these fundamental calcium-dependent mechanisms but exhibits a more pronounced early ionic response. In vitro studies have shown that Biodentine releases significantly higher calcium concentrations than several other calcium silicate-based materials during the early stages after placement [42,43]. This initial burst effect is attributed partly to CaCl_2_, which accelerates setting and calcium release and may promote signaling pathways involved in pulp cell differentiation.

Environmental scanning electron microscopy/energy-dispersive X-ray spectroscopy (ESEM/EDX) analyses have confirmed abundant calcium release from Biodentine accompanied by calcium/phosphate deposition on the material surface [41,43]. Development of this apatite-like layer may improve sealing, fill interfacial pores, and establish favorable conditions for reparative dentin formation. A Ca/P ratio of approximately 2.5 has been reported in these deposits, suggesting an initially irregular and relatively thin apatite coating associated with intensive calcium release [43]. Biodentine has additionally been reported to modulate transforming growth factor beta 1 (TGF-β1), thereby supporting odontoblast differentiation and hard tissue formation [42].

ERRM demonstrates formulation-dependent bioactivity associated with calcium ion release and apatite formation. In vitro comparisons demonstrated greater Ca^2+^ release and higher pH with the fast-setting ESF formulation over seven days, together with more pronounced hydroxyapatite formation following immersion in phosphate-buffered saline, compared with regular-setting ES [17]. However, these findings primarily reflect physicochemical bioactivity and do not directly establish differences in pulp-cell proliferation or tissue regeneration.

Histological evidence from a recent study comparing related TotalFill BC RRM formulations demonstrated greater dentin-bridge continuity after four weeks with Fast Set Putty than with the regular-setting Paste formulation [32]. Nevertheless, the limited sample size and short observation period restrict conclusions regarding long-term regenerative performance.

CEM cement differs from the other materials through its relatively high calcium, phosphorus, and silicon content [18]. Interactions between calcium and phosphate ions favor hydroxyapatite crystal formation [18,114], which has been demonstrated even in saline solutions [19]. Silicon contributes through formation of calcium silicate hydrate phases, while CEM cement simultaneously stimulates proliferation, attachment, and differentiation of dental pulp stem cells toward odontoblast-like phenotypes [18,19]. These observations support a potential role for selected CEM preparations in mineralization-related biological responses, but findings from experimental models and short-term human histological studies do not establish sustained tissue regeneration or clinical superiority [18,19,115].

Calcium release, alkalization, and apatite formation demonstrate physicochemical bioactivity but do not independently establish tissue regeneration or long-term clinical effectiveness. These outcomes require separate biological and clinical validation.

### 4.6. Antimicrobial Activity

Antimicrobial activity is relevant because persistent infection contributes to root canal treatment failure [114]. All four materials exhibit antibacterial activity, with magnitude and spectrum influenced by composition, alkalization, ion release, concentration, and test conditions.

MTA exhibits antibacterial and antifungal activity primarily as a result of environmental alkalization. After setting, MTA may reach a pH of approximately 12.5, which can disrupt microbial cell membranes and contribute to bactericidal activity [116]. Antibacterial activity has been reported against *Enterococcus faecalis* and *Streptococcus mutans* under selected experimental conditions. However, susceptibility is species- and assay-dependent, and one comparative agar-diffusion investigation found no inhibitory effect of the tested MTA formulation (Neo-Putty) against *Lactobacillus acidophilus* [116,117]. The combination of antibacterial activity and strong sealing ability is considered an important contributor to successful endodontic outcomes.

Biodentine demonstrates a broadly similar alkalization-dependent antimicrobial mechanism. Its pH may approach 12 during hydration, and significant inhibition has been reported against E. faecalis, Candida albicans, and S. mutans [53]. In some comparative studies, Biodentine demonstrated stronger antibacterial activity against E. faecalis than ERRM and MTA Plus [56]. These findings indicate substantial antimicrobial potential, although the magnitude of this effect in vivo should be interpreted cautiously because inhibition-zone assays and other in vitro methods do not reproduce the complex microbial and buffering conditions present within the root canal system.

ERRM also exhibits antibacterial activity related primarily to its strongly alkaline environment during hydration [45]. Its activity against E. faecalis has been reported to be comparable to that of MTA [10]. However, comparative studies suggest that its overall antibacterial and antifungal effects may be lower than those observed for some other calcium silicate-based materials. In the same experimental setting, Biodentine demonstrated the strongest antibacterial activity against E. faecalis, whereas MTA Plus showed the most pronounced antifungal effect against C. albicans [56].

CEM cement demonstrates substantial antimicrobial activity and, in several studies, has shown effects comparable to calcium hydroxide and greater than those observed for MTA and other calcium silicate cements [10,18,47]. Its antimicrobial activity is associated with calcium hydroxide formation during hydration of calcium silicate phases and the reaction of calcium oxide with water. Subsequent dissociation of calcium hydroxide releases calcium and hydroxyl ions, increasing local pH [18,19,47]. CEM may additionally contain diffusible inhibitory components with greater diffusion potential than those of some other calcium silicate-based materials [10,19,47].

Against E. faecalis, CEM cement has shown higher antibacterial activity than both MTA and Biodentine in some experimental models [10,18]. Its efficacy appears to increase with longer incubation and higher material concentration [19]. CEM has also demonstrated fungicidal activity against C. albicans, with complete killing reported within 24–48 h under selected experimental conditions [19]. Interestingly, the presence of dentin powder has been shown to enhance its antibacterial activity further. In aqueous conditions, CEM eliminated more than 95% of bacteria within 1 min in the presence of dentin [19].

Although all four materials demonstrate antimicrobial activity, differences in microbial strains, exposure times, material concentrations, and assay methods limit direct comparisons. In particular, findings from diffusion-based and direct-contact assays should not be interpreted as equivalent measures of antimicrobial effectiveness.

The principal biological and antimicrobial characteristics reported for the four materials are summarized in Table 3.

### 4.7. Tooth Discoloration

Tooth discoloration represents an important clinical limitation of selected calcium silicate-based cements, particularly when the material is used in esthetically relevant areas. The risk of discoloration is strongly influenced by material composition, especially the type of radiopacifier incorporated into the formulation.

MTA has historically been associated with the greatest discoloration potential among the materials considered in this review. White MTA, introduced in 2002, reduced this risk compared with gray MTA [119]. Nevertheless, an in vitro study using extracted human molars reported perceptible coronal discoloration with ProRoot MTA and MTA Angelus over an observation period of up to 2 years [120]. According to some studies, discoloration may become visible as early as 3 months after placement and may increase over time [119]. This effect is most likely related to the presence of bismuth oxide (Bi_2_O_3_), which under certain conditions, including light exposure or elevated temperature, may undergo reduction to metallic bismuth and contribute to dark staining of dentin [35].

Biodentine was developed without bismuth oxide and instead uses zirconium oxide as a radiopacifier. This compositional difference substantially reduces the discoloration potential compared with conventional MTA and represents an important clinical advantage in esthetically demanding procedures [10,11,12,13]. Its lower tendency to produce coronal staining should therefore be considered particularly relevant in anterior teeth and in vital pulp therapy procedures where the material is positioned close to the crown.

ERRM likewise avoids bismuth oxide and uses alternative radiopacifying components, including zirconium oxide and tantalum-containing compounds. Based on its formulation, ERRM would therefore be expected to carry a lower discoloration risk than conventional MTA. However, comparative long-term discoloration data remain less extensive than those available for MTA and Biodentine, and this potential advantage should therefore be interpreted cautiously.

CEM cement is also described as a tooth-colored material and has been associated with a lower risk of post-treatment discoloration than conventional MTA. However, the available comparative evidence is more limited than for MTA and Biodentine. Accordingly, although its composition and clinical appearance suggest favorable esthetic performance, additional long-term comparative studies are required to define its discoloration profile more precisely.

### 4.8. Clinical Handling

Clinical handling depends on several practical characteristics, including material consistency, mixing requirements, working time, delivery method, flowability, moisture sensitivity, washout resistance, and operator dependence. These characteristics may substantially influence clinical usability even when physicochemical and biological properties are otherwise favorable.

Conventional MTA formulations remain associated with several handling-related limitations. Their relatively prolonged setting time and difficult manipulation may complicate placement and increase susceptibility to displacement or washout before complete maturation. Some MTA formulations may also exhibit porosity and reduced physical stability under selected wet conditions. Recent investigations suggest that incorporation of 2% bioactive glass may improve some of these properties by accelerating apatite layer formation and enhancing sealing ability [33]. The relatively high price of MTA may additionally limit routine use. A single 0.5 g package has been reported to cost approximately €45 [13], which may represent a practical disadvantage compared with more affordable alternatives.

Biodentine generally provides more convenient handling than conventional MTA because of its shorter setting time and capsule-based preparation. This facilitates more standardized mixing and may reduce the need for prolonged protection of the material during early maturation. However, its clinical performance remains sensitive to environmental conditions. Conflicting results have been reported regarding washout resistance, particularly in surgical applications such as root-end filling, and bond strength may decrease in the presence of blood contamination. These findings indicate that although Biodentine offers important practical advantages, careful moisture and bleeding control remain clinically relevant.

ERRM differs fundamentally from conventional powder–liquid hydraulic cements because it is delivered as a ready-to-use premixed material. This reduces mixing-related operator variability during preparation and contributes to improved clinical handling [14,15,16,30]. Premixed delivery also facilitates direct placement and reduces the risk of inconsistencies in the powder-to-liquid ratio. However, because ERRM is moisture-activated, its setting behavior remains dependent on environmental conditions and local moisture availability.

Handling characteristics of CEM-related materials are formulation-dependent. In the study by Asgary et al. [49], the experimental NEC formulation exhibited greater flow (14 ± 1 vs. 10 ± 0.79 mm) and lower film thickness (174 ± 25 vs. 452 ± 63 µm) than tooth-colored ProRoot MTA under the reported testing conditions. However, its Methods specify three measurements per material, while Table 2 gives n = 5 for NEC and n = 3 for MTA; the sample-count discrepancy remains unresolved [49]. These experimental NEC findings should not be generalized to commercial CEM formulations. Conventional CEM preparations have also been described as relatively easy to manipulate, although mixing and environmental conditions influence their handling and setting [18,19,48,54].

## 5. Clinical Translation: Matching Material to Clinical Indication

The clinical value of calcium silicate-based cements depends not only on their physicochemical and biological properties, but also on whether these characteristics address the specific requirements of a given procedure. Material selection should therefore be considered in the context of the biological and technical demands of the indication, local tissue conditions, handling requirements, and the maturity of the supporting evidence. Accordingly, the following sections evaluate the evidence by clinical indication, considering baseline pulpal diagnosis, study design, sample size, treatment protocol, follow-up duration, and the presence of an appropriate comparator. Clinical success, radiographic outcomes, and histological findings are interpreted as distinct, study-defined endpoints. Clinical success may involve the absence of symptoms and maintenance of pulp vitality, where applicable, whereas radiographic assessments concern findings such as periapical healing or continued root development. Histological outcomes, including inflammatory responses and dentin-bridge formation, provide tissue-level information but do not independently establish long-term clinical success or complete tissue regeneration. Findings from laboratory and animal studies are considered supportive preclinical evidence rather than equivalent measures of clinical effectiveness.

The principal clinical and human histological evidence supporting these indication-specific comparisons is summarized in Table 4, with particular attention to study design, population, follow-up duration, and methodological limitations.

### 5.1. Vital Pulp Therapy

Vital pulp therapy aims to preserve pulp vitality and support tertiary dentin formation in teeth affected by trauma, caries, or pulp exposure [3,44]. The principal clinical requirements are therefore biological compatibility with pulp tissue, control of bacterial contamination and inflammation, establishment of a stable seal, and support of reparative dentinogenesis.

Among the materials considered in this review, MTA has the most mature clinical evidence and remains the reference material for direct pulp capping and pulpotomy. Its clinical use is supported by favorable sealing, biocompatibility, alkalization, and mineralization-related activity [3]. MTA also interacts with dentin through physical adaptation and interfacial mineral deposition, which may contribute to long-term sealing and tissue repair.

Biodentine is likewise supported by a substantial clinical evidence base in vital pulp therapy and has been used in immature and mature permanent teeth [3]. Its principal practical advantages are shorter setting time, easier handling, and lower discoloration potential. Several studies have reported favorable dentin bridge formation and pulp responses, although comparative findings versus MTA are not uniform. Some studies suggest reduced inflammation or thicker dentin bridges, whereas others report greater pulpal inflammation. Claims of superiority over MTA should therefore be made cautiously [3,44].

CEM cement has also been investigated in vital pulp therapy, including direct and indirect pulp capping and partial or full pulpotomy in primary and permanent teeth [10,18,19]. In a 24-month split-mouth randomized clinical trial involving 102 primary second molars in 51 children, CEM cement was compared with electrosurgery followed by zinc oxide–eugenol placement (ES/ZOE). Clinical success was 100% in both groups, whereas radiographic success was 90% for CEM and 95.2% for ES/ZOE, without a statistically significant difference (*p* = 0.625) [20]. These findings support the clinical potential of CEM in primary molar pulpotomy but should not be generalized to other indications or formulations. Favorable outcomes have also been reported in permanent molars with irreversible pulpitis [19]. Histological investigations have demonstrated dentin-bridge formation and variable inflammatory responses, while selected studies suggest direct pulp-capping outcomes comparable to MTA [19,115].

ERRM has also been investigated as a direct pulp-capping material. In a comparative clinical study involving 60 permanent posterior teeth, Biodentine and ERRM demonstrated clinical success rates of 78.6% and 64.7%, respectively, after 12 months, without a statistically significant difference between groups [45]. The absence of a statistically significant difference should not be interpreted as evidence of equivalence, particularly given the limited sample size and follow-up duration of this individual clinical study. Additional preclinical evidence from a rabbit model demonstrated favorable histological responses following ERRM application, particularly when combined with low-level laser therapy [46]. This study included 24 teeth from six experimental rabbits, evaluated after 2 weeks or 2 months, with two additional rabbits serving as untreated controls. The experimental comparison was ERRM with versus without laser therapy, rather than ERRM versus another cement [46]. However, these experimental findings should not be interpreted as direct evidence of clinical effectiveness in humans. Overall, the clinical evidence supporting ERRM remains less extensive than that available for MTA and Biodentine.

### 5.2. Apexogenesis and Apexification

Management of permanent teeth with incomplete root development is complicated by open apices, wide canals, and thin dentinal walls. In apexification, the principal clinical requirement is formation of a stable apical barrier that permits predictable obturation, whereas in apexogenesis the objective is preservation of pulp vitality to allow continued root development and dentinal wall thickening.

MTA has an established clinical role in apexification, particularly as an apical barrier in immature permanent teeth with necrotic pulps [5,121]. In a long-term clinical case series involving necrotic immature permanent incisors treated with an MTA apical barrier and adhesive restoration, clinical and radiographic healing was reported in 66 of 69 recalled teeth (96%) after 5–15 years of follow-up, with a mean observation period of 8.29 years [122]. However, only 69 of the original 83 treated teeth were available for recall, and the noncomparative design does not establish superiority over alternative apical barrier materials. Furthermore, treatment outcomes reflect the combined effects of the MTA barrier and subsequent restorative procedures. Although an MTA apical plug facilitates subsequent root canal obturation, apexification does not promote continued root development, and treated immature teeth may remain susceptible to cervical or root fractures [121].

In apexogenesis, MTA supports reparative dentin formation, odontoblast differentiation, continued root wall thickening, and apical closure [5,11,35]. Depending on the extent of pulpal inflammation, the procedure may involve direct pulp capping, partial pulpotomy, or total pulpotomy while preserving radicular pulp vitality [5,11].

Biodentine represents an alternative to MTA when shorter setting time, favorable handling, and reduced discoloration risk are clinically relevant. Its ability to promote calcium phosphate and hydroxyapatite-like deposition has been demonstrated experimentally [43]. A systematic review and meta-analysis focused on apexogenesis in young permanent mandibular molars included six trials, although their populations and procedures varied. Its pooled radiographic comparisons found no statistically significant difference between Biodentine and MTA at six and twelve months [11]. However, the limited number of studies and methodological variability do not establish clinical equivalence or long-term superiority.

CEM cement has also been used for apexification, apical plug formation, and apexogenesis [10,18,19]. However, comparative clinical evidence remains less extensive than for MTA and Biodentine. Evidence for ERRM in these indications is comparatively limited within the studies considered in this review and does not currently support an equally mature clinical position.

Thus, when a predictable apical barrier and the most mature clinical evidence are the principal priorities, MTA remains the most extensively supported option. Biodentine provides a reasonable alternative when faster setting and reduced discoloration are important, particularly in apexogenesis. CEM cement may also be considered in selected cases, but its comparative clinical evidence is less mature, while the role of ERRM requires further clinical validation.

### 5.3. Perforation Repair

Root perforation creates an artificial communication between the root canal system and surrounding periodontal tissues and increases the risk of microbial contamination of periradicular structures [123]. Successful repair therefore requires rapid and stable sealing, resistance to displacement, dimensional stability, and biological compatibility under conditions that are frequently complicated by blood contamination and local inflammation.

MTA has a comparatively established clinical evidence base for root perforation repair, supported by its sealing properties and biological compatibility [123,124]. In a prospective longitudinal cohort of 124 patients, initial healing was observed in 115 cases within the first two years, although 48 subsequent recurrences occurred during extended follow-up [125]. These findings demonstrate that favorable initial outcomes do not necessarily indicate long-term stability. Furthermore, as this was a single-arm cohort, the results cannot establish superiority over other repair materials. Perforation size, location, and positive pre-treatment periodontal probing were among the factors associated with treatment outcomes [125].

Biodentine has demonstrated favorable marginal adaptation and bond strength in perforation-repair models. However, blood contamination may reduce its adhesion relative to MTA [3]. Its practical value may therefore be greatest when adequate bleeding control can be achieved and rapid material stabilization is desirable.

In an in vitro furcation-perforation study, EndoSequence Root Repair Material was compared with EndoSeal MTA, a root canal sealer, under conditions with and without Chloroquick irrigation [126]. Push-out bond-strength findings from this study are specific to the tested formulations and experimental conditions. They do not establish superiority over conventional MTA repair cements or predict clinical outcomes following perforation repair.

CEM cement has also been used in root and furcation perforation repair [18,19]. Its sealing properties have been reported as broadly comparable to those of MTA in selected experimental models [19,65], although long-term comparative clinical evidence remains less extensive.

Accordingly, perforation repair should be guided primarily by the need for a durable seal under potentially contaminated conditions. MTA remains the most evidence-supported material when long-term clinical experience is prioritized. Biodentine, ERRM, and CEM cement provide potentially useful handling, bonding, or sealing characteristics, but their selection should depend on bleeding control, defect characteristics, and the maturity of indication-specific evidence rather than on assumed overall superiority.

### 5.4. Root-End Filling

Root-end filling is used during surgical endodontic treatment when orthograde treatment is not feasible or has failed [3,5]. The principal requirements in this indication are stable sealing, resistance to washout, dimensional stability, and favorable interaction with periapical tissues in a moist surgical environment.

MTA is among the most extensively investigated root-end filling materials. A systematic review and network meta-analysis including randomized and observational clinical studies ranked MTA favorably for clinical and radiographic success; however, methodological differences and uncertainty in the comparative estimates limit conclusions regarding its superiority over alternative materials [127]. Laboratory investigations also support its sealing and washout resistance under selected conditions, but these endpoints should not be considered direct predictors of long-term surgical success [12].

Biodentine has demonstrated favorable sealing properties in laboratory models and promising tissue responses in selected preclinical studies summarized in the literature [3]. Nevertheless, experimentally observed washout resistance is variable, with some studies reporting substantial material loss shortly after placement [12]. These findings indicate that its performance is sensitive to the experimental environment and do not establish superior clinical outcomes.

ERRM offers practical advantages associated with premixed delivery. Its resistance to material loss has been investigated in simulated surgical models [128], but these experimental observations do not establish improved long-term healing compared with MTA. Similarly, CEM cement has demonstrated potentially favorable sealing and bioactive properties, although the available clinical comparisons remain limited and much of the evidence is derived from laboratory investigations or secondary reviews [18,19].

### 5.5. Complete Orthograde Obturation

Complete orthograde obturation should be clearly distinguished from localized applications of calcium silicate-based cements, such as apical plugs or barriers used in apexification and perforation repair. In apexification, a short apical barrier may be formed using MTA or another calcium silicate-based material, while the remaining canal is subsequently obturated using conventional filling materials [3,121]. Such localized applications do not constitute evidence supporting complete orthograde obturation with the same cement.

Conventional gutta-percha combined with an endodontic sealer remains the established approach for routine root canal obturation. Reported clinical success depends on baseline diagnosis, treatment protocol, and follow-up duration [3]; findings from heterogeneous clinical settings should not be interpreted as direct comparative evidence against complete orthograde obturation with calcium silicate-based cements. MTA has also been used for this purpose in selected cases, particularly in immature teeth. However, laboratory findings concerning sealing ability and fracture resistance, together with limited clinical observations, do not establish its superiority or routine suitability for this application [3,119,121].

Biodentine has demonstrated favorable sealing properties in selected experimental models, particularly when used as an apical barrier, but these findings do not establish its effectiveness as a routine material for complete orthograde obturation [119]. CEM cement has likewise been investigated in selected bio-obturation applications, with supporting evidence derived largely from experimental studies and secondary reviews [19,65]. Its use as a localized coronal plug during internal bleaching was evaluated in an in vitro bacterial-microleakage study [129]. Clinical evidence for ERRM as a complete orthograde obturation material remains insufficient and should not be conflated with studies involving other premixed bioceramic sealers.

Therefore, the evidence supporting localized use of calcium silicate-based cements should not be extrapolated to complete orthograde obturation. Long-term sealing, retreatability, and patient-level outcomes require separate clinical evaluation. Current evidence does not justify routine replacement of conventional gutta-percha-based obturation with the four materials considered in this review.

### 5.6. Regenerative Endodontic Procedures

Regenerative endodontic procedures aim to promote continued root development and restoration of biological function in immature teeth with necrotic pulp. In this context, the material should provide a biologically compatible coronal or intracanal barrier without compromising stem/progenitor cell activity, vascularization, or tissue organization. However, the outcome of regenerative treatment depends on the entire protocol, including disinfection, scaffold formation, vascular support, and recruitment of endogenous progenitor cells.

MTA has been used in regenerative endodontic procedures, particularly as a coronal barrier, owing to its sealing and biocompatibility characteristics [3]. In a separate in vitro investigation, polycaprolactone/chitosan scaffolds enriched with MTA increased the expression of selected angiogenesis-related markers, including VEGFR-2, Tie2, and angiopoietin-1, in human dental pulp stem cells [130]. However, these findings relate to a composite scaffold system rather than unmodified MTA cement and do not demonstrate successful clinical revascularization or pulp regeneration. The contribution of MTA should therefore be considered within the broader regenerative protocol, including disinfection, scaffold formation, and vascular support.

Biodentine has also been investigated as a coronal barrier in regenerative endodontic procedures. In a randomized clinical trial involving 26 necrotic immature permanent anterior teeth, Aly et al. [131] compared Biodentine and white MTA as coronal barrier materials following a standardized revascularization protocol. After 12 months, no statistically significant differences were found in clinical success or root-length increase. However, tooth discoloration occurred in one Biodentine-treated tooth compared with seven MTA-treated teeth (*p* = 0.01). The limited sample size and follow-up duration do not establish clinical equivalence or superior regenerative efficacy. Biodentine has also been incorporated into protocols using platelet concentrates and stem cell-based adjuncts [3], although outcomes from these combined interventions cannot be attributed to the cement alone.

CEM cement has also been used in regenerative procedures and in revascularization of necrotic immature permanent molars [10,18,19]. These reports suggest potential clinical utility, although the comparative evidence remains less extensive than for MTA and Biodentine.

Evidence for ERRM in regenerative endodontic procedures remains limited within the studies considered in this review, despite favorable biological and handling characteristics demonstrated in experimental models.

**Figure 3:**
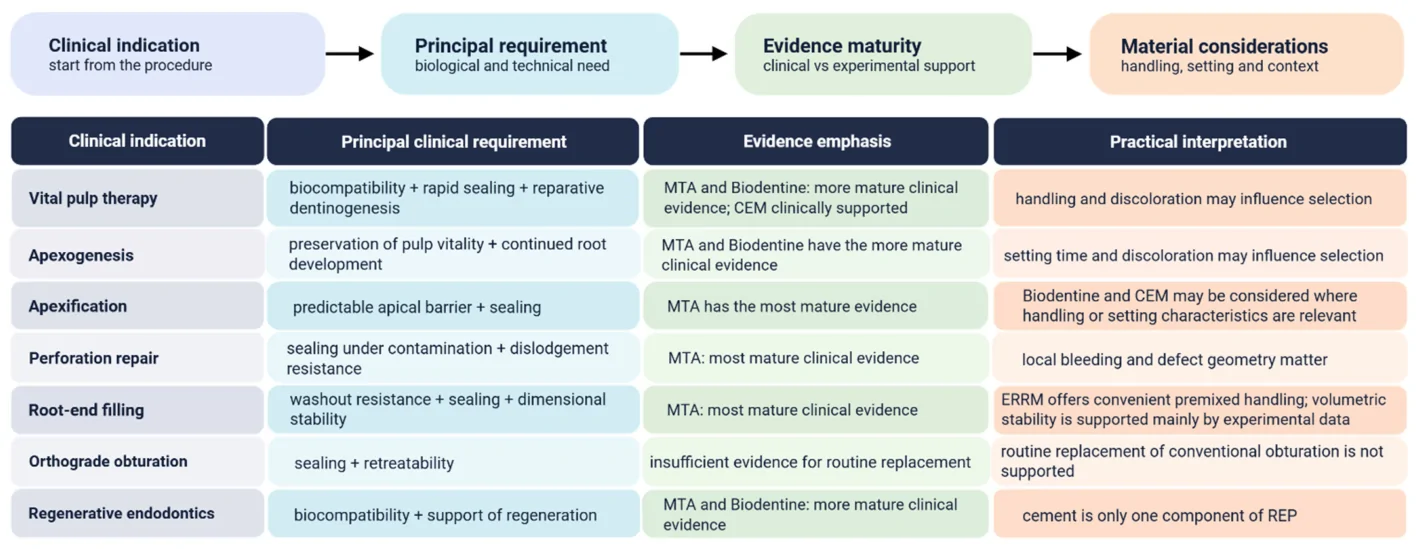
Indication-specific framework for the clinical selection of calcium silicate-based endodontic cements. Material selection should begin with the biological and technical requirements of the clinical indication and subsequently consider the maturity of supporting evidence and formulation-specific practical characteristics. The framework summarizes the principal considerations for MTA, Biodentine, ERRM, and CEM cement and is intended to support indication-specific interpretation rather than establish a universal hierarchy of materials. Evidence maturity differs between indications, and a material that is well supported in one clinical context may remain supported predominantly by experimental or limited clinical data in another. Created in BioRender. Rozpędek-Kamińska, W. (2026) https://BioRender.com/kpdm6q4, accessed on 1 October 2026.

Clinical evidence for MTA and Biodentine in regenerative endodontic procedures primarily concerns their use as coronal barriers [3], whereas evidence for CEM cement and ERRM remains more limited [10,18,19]. Regenerative outcomes depend on the complete treatment protocol, including disinfection, scaffold formation, vascular support, and case selection. Figure 3 summarizes the relationship between clinical requirements, evidence maturity, and material selection.

**Table 4:** Indication-Specific Clinical and Human Histological Evidence for Selected Calcium Silicate-Based Cements.

Indication/Study	Population, Tooth Type, and Sample Size	Procedure and Comparator	Follow-Up	Principal Outcomes	Main Limitations
Vital pulp therapy–Systematic review and meta-analysis [2]	40 RCTs; 1701 patients, 3168 primary and permanent teeth	MTA, Biodentine, and other CSCs	Selected comparisons at 6–12 months	MTA and Biodentine showed broadly comparable clinical and radiographic success	Heterogeneous procedures, outcome definitions, and follow-up periods
Primary molar pulpotomy–Split-mouth RCT, Khorakian et al. [20]	102 primary second molars in 51 children aged 4–6 years; 51 teeth per group at baseline, with 40 CEM and 42 ES/ZOE teeth evaluated at 24 months	CEM pulpotomy vs. electrosurgery followed by zinc oxide–eugenol (ES/ZOE); all teeth restored with preformed metal crowns.	24 months	Clinical success: 100% in both groups; radiographic success: 90% for CEM vs. 95.2% for ES/ZOE (*p* = 0.625)	Loss to follow-up; primary second molars only; procedural rather than material-only comparator; limited generalizability
Direct pulp capping–Comparative clinical study [45]	60 permanent posterior teeth with deep caries and vital pulps, without signs of irreversible pulpitis or periapical disease; 30 teeth per group at baseline.	Biodentine vs. ERRM	12 months	Clinical success: 78.6% vs. 64.7%; difference not statistically significant	Limited sample size, loss to follow-up, and lack of long-term evaluation
Direct pulp capping–Human histological study [115]	40 healthy, caries-free permanent premolars scheduled for orthodontic extraction; 38 analyzed (9 MTA, 9 CEM, 10 Biodentine, and 10 untreated controls).	MTA vs. Biodentine vs. CEM cement, with untreated controls	6 weeks	Biodentine showed greater inflammation than MTA and CEM; dentin-bridge formation was broadly comparable	Experimentally induced pulp exposure in initially healthy teeth; extraction and histological examination after 6 weeks; no assessment of long-term clinical success.
Apexogenesis–Systematic review and meta-analysis [11]	6 clinical trials focused on immature permanent mandibular molars; populations and vital pulp therapy procedures varied	Biodentine vs. MTA and other calcium-based materials	6–12 months in principal comparisons	Pooled radiographic comparisons found no significant difference between Biodentine and MTA at 6 and 12 months	Limited number of trials; heterogeneous populations, procedures, and outcomes
Apexification–Long-term clinical case series, Ree and Schwartz [122]	83 necrotic immature permanent incisors in 72 patients at baseline; 69 teeth in 60 patients available for recall	MTA apical barrier followed by adhesive restoration, with or without fiber posts; no material comparator	5–15 years (mean 8.29 years)	Clinical and radiographic healing in 66/69 recalled teeth (96%)	Noncomparative case series; incomplete recall; outcomes also influenced by the restorative protocol
Perforation repair–Prospective clinical cohort [125]	124 patients with root perforations	MTA; no concurrent comparator	Up to 14 years	Initial healing in 115/124 cases within 2 years; 48 subsequent recurrences among initially healed cases during extended follow-up	Single-arm design; substantial late recurrence; absence of a comparative treatment group
Root-end filling – Systematic review and network meta-analysis [127]	16 studies; 2391 patients across 15 studies contributing to the 12-month analysis	MTA, RRM, Super EBA, IRM, and gutta-percha	At least 12 months; selected longer follow-up	MTA ranked highest in the principal analysis; sensitivity analyses favored MTA and selected alternatives	Heterogeneous study designs, network inconsistency, and limited long-term evidence
Regenerative endodontic procedures–Randomized clinical trial, Aly et al. [131]	26 necrotic immature permanent anterior teeth; 13 teeth per group	Revascularization protocol using Biodentine vs. white MTA as coronal barrier materials	12 months	No significant difference in clinical success or root-length increase; discoloration in 1 Biodentine vs. 7 MTA cases (*p* = 0.01)	Small sample size; limited follow-up; outcomes reflect the entire regenerative treatment protocol

RCT, randomized controlled trial; CSC, calcium silicate-based cement; MTA, mineral trioxide aggregate; CEM, calcium-enriched mixture; ERRM, EndoSequence Root Repair Material; RRM, root repair material; IRM, intermediate restorative material. Study populations and tooth types are specified separately. Human histological responses should not be interpreted as equivalent to long-term clinical effectiveness.

## 6. Discussion

### 6.1. What Has Improved Beyond MTA?

The mechanistic-to-clinical sequence illustrated in Figure 1 is proposed as a conceptual framework for organizing the evidence, rather than a validated causal pathway. Available studies typically assess formulation, physicochemical behavior, cellular responses, and clinical outcomes separately; none of the investigations discussed here establishes the entire sequence from cement hydration to sustained clinical success.

Direct head-to-head studies identify trade-offs rather than uniform improvements in material performance. Biodentine set earlier than ProRoot MTA in one experimental protocol but exhibited greater mass loss after prolonged immersion and lower radiopacity [50]. The clinical relevance of these differences depends on the indication, as setting time, dissolution, and radiographic visibility reflect different aspects of material performance. Similarly, greater calcium release and apatite formation by ESF compared with ES [17] cannot explain differences in short-term dentin-bridge continuity observed in a separate study of related TotalFill formulations [32]. These products and outcomes were not evaluated within a linked experimental model. Establishing whether altered hydration or ion release contributes to improved healing requires matched physicochemical and tissue-level measurements, followed by indication-specific clinical validation [4,6,76].

### 6.2. Physicochemical–Biological Trade-Offs

Radiopacifier chemistry illustrates why modification of one material property may generate competing effects. A primary laboratory investigation detected bismuth release from ProRoot MTA and reduced stromal-cell viability under defined exposure conditions [75]; broader evidence supports considering radiopacifier composition when assessing material-related biological responses [75,76]. However, these findings do not demonstrate that replacing bismuth oxide with zirconium- or tantalum-based compounds improves clinical healing.

An unresolved question is whether laboratory measurements of dissolution and pore structure reliably predict sealing durability or biological performance. Biodentine dissolution varies with immersion duration and acidity [76,132], while the surrounding medium can modify surface phases and material maturation [76,133,134]. Porosity measurements obtained using immersion-based methods [41] and micro-CT [61] are likewise not directly comparable. In a direct micro-CT investigation of furcation-perforation repair materials, Yalniz et al. [88] found the lowest total porosity with EndoSequence Root Repair Material, whereas BioAggregate showed the lowest open porosity. These results demonstrate that distinct porosity parameters should not be treated as interchangeable measures of material performance. However, sealing ability, mechanical strength, and clinical healing were not directly evaluated in that study. Whether specific pore characteristics predict fluid transport, material degradation, or interfacial failure therefore remains unresolved and requires paired microstructural and functional assessments under comparable experimental conditions.

### 6.3. Laboratory Performance Versus Clinical Performance

Laboratory endpoints cannot be treated as clinical surrogates until their predictive value has been established. In an extracted-tooth model, fluid-filtration leakage differed over time between Biodentine and ProRoot MTA [62], but these findings do not demonstrate corresponding differences in long-term periapical healing. Moreover, fluid-filtration, dye-penetration, and bacterial-leakage assays assess distinct phenomena [135]. Similarly, acidic conditions affect material dissolution and maturation in experimental models [132,133,134], whereas the clinical environment involves changing inflammatory, microbial, and mechanical influences. The central unresolved issue is whether measurable differences in laboratory performance predict clinically meaningful outcomes independently of baseline diagnosis, procedural factors, contamination, and restoration quality.

Cell-based findings also require endpoint-specific interpretation. Static culture systems do not reproduce the cellular architecture of the dentin–pulp complex, and ex vivo human tooth models can provide more tissue-level information [23]. Immunomodulatory responses and ion-related bioactivity may be relevant to repair [23,111], but neither alone demonstrates organized, durable tissue regeneration. Short-term human histological observations [32,115] remain distinct from clinical treatment success, and angiogenic-marker changes observed with MTA-enriched polymeric scaffolds [130] should not be attributed to unmodified MTA. Likewise, laboratory strength tests generally use loading and conditioning protocols unlike repeated functional stresses in vivo [6,76]. This requires studies linking standardized physicochemical, cellular, and tissue-level measurements with predefined, indication-specific clinical and radiographic outcomes.

Representative formulation-specific molecular and cellular findings are summarized in Table 5. These studies identify distinct experimental responses involving inflammatory signaling, mineralization-related gene expression, growth factor regulation, and cell survival. However, the strength of mechanistic evidence differs across the investigated materials. Changes in transcript abundance or protein expression generally demonstrate associations rather than causal pathway activation, whereas experiments involving pathway inhibition provide more direct functional evidence. Importantly, these observations remain dependent on the cellular model, material preparation, exposure conditions, and experimental endpoint and cannot independently establish regeneration of the human dentin–pulp complex.

The relationship between molecular responses and tissue repair remains incompletely established. For example, MTA and CEM cement produced different growth factor expression profiles in hDPSCs [136], while another comparative investigation reported relatively limited odontogenic marker expression following CEM exposure [138]. Such findings highlight the influence of experimental context rather than establishing a consistent molecular hierarchy between materials. Similarly, ERK-dependent cell survival observed with ERRM and MTA [137] and angiogenesis-related marker expression induced by MTA-containing scaffolds [130] do not demonstrate functional pulp regeneration. Establishing these relationships requires pathway-specific functional experiments, appropriate tissue-level models, and clinical validation.

### 6.4. Heterogeneity of Experimental Methodology

**Figure 4:**
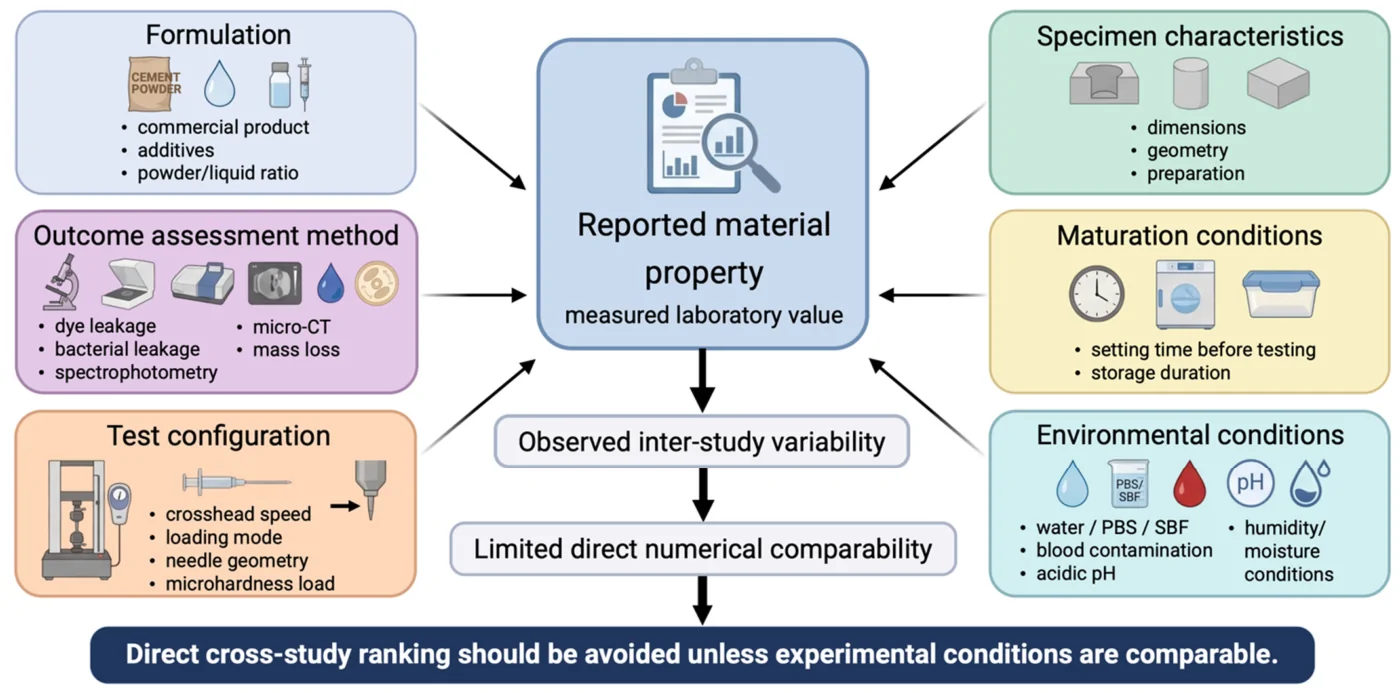
Major sources of methodological heterogeneity in studies of calcium silicate-based endodontic cements. Differences in formulation, specimen characteristics, maturation and environmental conditions, test configuration, and outcome assessment can substantially influence measured laboratory values. These variables contribute to inter-study variability and limit direct numerical comparison across independent studies. Created in BioRender. Rozpędek-Kamińska, W. (2026) https://BioRender.com/kpdm6q4, accessed on 1 October 2026.

Apparently contradictory findings cannot be interpreted without first establishing whether the underlying studies evaluated comparable endpoints. For example, the two- and seven-day intervals reported for CEM solubility represent different maturation periods before an identical immersion test [68], unlike studies using longer immersion protocols [50,82]. Similarly, marginal-gap measurements obtained from a single-tooth SEM model [59] and a larger simulated immature-tooth model [60] involve different sampling units and anatomical configurations. Differences in conditioning, drying procedures, test media, and leakage definitions further complicate comparisons [6,41,70,76,80,135]. Such discrepancies cannot be resolved by combining published numerical values. Replicated head-to-head investigations should use clearly identified commercial formulations, matched specimen dimensions, standardized maturation and exposure conditions, consistent endpoints, and predefined statistical analyses. Supplementary Table S1 and Figure 4 identify the methodological variables that should be documented to distinguish genuine material-related differences from experimental variability.

### 6.5. Strength and Maturity of Clinical Evidence

Clinical studies illustrate a further limitation: favorable early outcomes do not necessarily predict sustained treatment success. In a comparative direct pulp-capping study, the absence of a statistically significant difference between Biodentine and ERRM does not establish clinical equivalence [45]. Similarly, initial healing following MTA perforation repair was followed by late recurrences in a single-arm longitudinal cohort, preventing conclusions about long-term comparative superiority [125]. These findings highlight the need for adequately powered, indication-specific comparative studies with clearly defined baseline diagnoses, standardized treatment protocols, separate clinical and radiographic endpoints, and follow-up periods sufficient to identify delayed failures or recurrence.

The rabbit study of ERRM with or without laser therapy [46] and short-term human histological investigations [32,115] provide tissue-level evidence but cannot substitute for controlled assessments of patient-level outcomes. Differences in patient selection, outcome definitions, and follow-up duration across clinical studies further complicate comparisons and should be considered when interpreting the available evidence [2,9,76].

The maturity of clinical evidence also differs across indications. For vital pulp therapy, MTA and Biodentine are supported by a broader comparative evidence base, including randomized trials and their systematic synthesis, whereas evidence for CEM cement and ERRM is based on fewer direct clinical comparisons [2,20,45]. In apexogenesis, available comparisons between Biodentine and MTA involve a limited number of heterogeneous clinical trials with relatively short follow-up [11]. For apexification, long-term follow-up of immature permanent incisors treated with an MTA apical barrier has been reported in a noncomparative case series [122], supporting clinical feasibility but not demonstrating comparative superiority. For perforation repair, long-term data support the clinical use of MTA but derive partly from single-arm investigations that cannot establish comparative superiority [125]. Root-end filling has been evaluated through network meta-analysis, although differences in study design and inconsistency between comparisons limit the certainty of material rankings [127]. For regenerative endodontic procedures, a small randomized trial comparing Biodentine with white MTA as coronal barrier materials provides direct but short-term clinical evidence [131], which should be distinguished from histological and laboratory findings [3,32]. These differences are considered qualitatively rather than through a formal certainty-grading system.

An additional limitation is the exclusion of other contemporary calcium silicate-based formulations, such as NeoMTA Plus, MTA Repair HP, and Bio-C Repair, from the primary comparative analysis [38,76,112]. Consequently, the conclusions are restricted to the selected materials, their specific formulations, and the clinical indications examined. They should not be extrapolated to all commercially available calcium silicate-based cements or interpreted as a comprehensive ranking of current endodontic materials.

## 7. Conclusions

The available evidence does not support a universal hierarchy among MTA, Biodentine, ERRM, and CEM cement. Direct comparative investigations identify formulation-dependent advantages and physicochemical trade-offs [17,32,50,75], whereas the strength of clinical evidence differs substantially across endodontic indications. The proposed mechanistic-to-clinical framework should therefore be regarded as a conceptual synthesis rather than evidence that improved setting kinetics, ion release, apatite formation, or cellular signaling predicts sustained clinical healing. Future research should integrate standardized, formulation-specific physicochemical and molecular assessments with prospective clinical investigations using clearly defined clinical and radiographic outcomes. Establishing these relationships is essential for determining which material properties have genuine prognostic and therapeutic relevance.

## 8. Future Perspectives

Further development of these materials should also address formulation-related limitations, particularly discoloration, radiopacity, handling, solubility, and mechanical stability, while preserving their favorable bioactivity. Optimization of radiopacifier composition may be particularly relevant for reducing discoloration and potential formulation-associated biological effects without compromising radiographic detectability.

Stimuli-responsive or adaptive materials capable of modifying ion release or antimicrobial activity in response to changes in the local environment represent a potential future direction. However, such approaches remain largely conceptual or experimental and require substantial preclinical validation before their clinical relevance can be established. Ultimately, advances in material design and stronger clinical evidence may allow more indication-specific selection of calcium silicate-based cements according to local biological conditions, treatment requirements, and patient-related factors.

## Figures and Tables

**Table 3:** Comparative biological response and antimicrobial profile of MTA formulations, Biodentine, CEM cement, and ERRM formulations.

Biological Feature	MTA Formulations	Biodentine	CEM Cement	ERRM Formulations
Cytocompatibility	Generally high viability in periodontal ligament fibroblasts and bone marrow–derived stem cells; slight apoptosis reported in some models [69,107,108]	Generally favorable cytocompatibility in monocyte/macrophage, hDPSC and fibroblast models [108,109,110]	Good viability in fibroblasts, hDPSCs and SCAP; set material generally more cytocompatible than freshly mixed cement [10,18,19]	Cytotoxicity generally comparable to MTA across several fibroblast and osteoblast-like cell models [10]
Inflammatory response	Increased IL-8 mRNA expression after 24 and 72 h and IL-6 expression after 72 h in hDPSCs; associated changes in S100A family gene expression [7].	Context-dependent response: increased IL-1α in a 3D fibroblast model, but downregulation of pro-inflammatory mediators including IL-8 in macrophage/hDPSC models [110,111]	Direct comparative cytokine data remain limited in the studies included in this review	Direct comparative cytokine data remain limited in the studies included in this review
Genotoxicity/formulation-associated safety	No major genotoxicity in some cellular models; bismuth oxide may contribute independently to cytotoxicity and other formulation-related concerns [75,108]	No significant genotoxicity reported in the evaluated monocyte/macrophage model; zirconium oxide avoids bismuth-related concerns [108]	Ames test data support a non-mutagenic profile, although concentration-dependent genotoxic effects have been reported in L929 cells [18]	The tested EndoSequence BC Root Repair formulation showed greater micronucleus formation than MTA Angelus and MTA Repair HP after 7 days in MC3T3 cells [112]
Biomineralization/regenerative activity	Sustained calcium release, apatite formation and mineralization-related signaling support hard-tissue barrier formation [41,42,113]	Pronounced early calcium release, Ca/P deposition and modulation of mineralization-related pathways support rapid interfacial bioactivity [41,42,43]	Calcium-, phosphate- and silicon-related activity promotes hydroxyapatite formation and odontogenic/osteogenic responses [18,19,114]	ESF showed greater calcium release and hydroxyapatite formation than ES in vitro [17]; TotalFill Fast Set Putty demonstrated greater dentin-bridge continuity than Paste in a short-term human histological study [32].
Antibacterial activity	Activity against *E. faecalis* and *S. mutans* under selected conditions [116,117]; no inhibition of *L. acidophilus* by Neo-Putty in an agar-diffusion study [117].	Strong activity against E. faecalis in several in vitro studies; activity is largely related to alkalization [53,56,118]	Pronounced antibacterial activity against E. faecalis in several experimental models; activity may exceed MTA under selected conditions [10,18,47]	Antibacterial activity broadly comparable to MTA in some studies, but lower than Biodentine in selected head-to-head experiments [10,56]
Overall interpretation	Most established biological benchmark, with extensive experimental and clinical experience	Strong early bioactivity and favorable cytocompatibility, with context-dependent inflammatory effects	Favorable biological and antimicrobial profile, but evidence remains less extensive and more heterogeneous than for MTA	Promising biological profile, particularly for premixed formulations, but clinical evidence remains less mature

**Table 5:** Representative molecular and cellular findings associated with selected calcium silicate-based endodontic materials.

Material/Formulation	Experimental Model	Exposure Conditions	Molecular Endpoints and Principal Findings	Ref.
White ProRoot MTA and Biodentine	Human dental pulp stem cells (hDPSCs)	Conditioned media at 0.002, 0.02, and 0.2 mg/mL; gene expression at 24 and 72 h	Concentration- and time-dependent S100A expression; increased IL-8 mRNA at 24 and 72 h and IL-6 at 72 h. Mineralization-related responses were assessed in separate assays.	[7]
Biodentine	LPS-activated THP-1-derived macrophages and hDPSCs	LPS (1 µg/mL) for 24 h, followed by Biodentine (2 mg/mL) for 24 h	Reduced IL-1β, IL-6, and IL-8 gene expression, with increased IL-10 and TGF-β expression. Cytokine secretion did not uniformly correspond to transcript levels.	[111]
MTA and CEM cement (investigated formulations)	Human dental pulp stem cells	Direct culture on prepared material discs for up to 14 days	Both materials increased DSPP and DMP1 expression relative to growth-medium controls; mineral deposition was assessed separately. TGF-β1 expression was greater with MTA, whereas BMP-2 and FGF4 expression was greater with CEM; ELISA findings supported the gene-expression results.	[136]
EndoSequence Root Repair Material and gray ProRoot MTA	Human dental pulp stem cells, periodontal ligament stem cells, and bone marrow mesenchymal stem cells	Material-coated surfaces; proliferation at 1, 3, and 5 days; survival under serum deprivation (0.2% or 0.5% serum)	Increased ERK phosphorylation and cell survival. Blocking ERK signaling with U0126 reduced material-associated survival, supporting pathway involvement in this cellular response.	[137]
Biodentine, CEM cement (BioniqueDent), and OrthoMTA	Human dental pulp stem cells	Conditioned media at 10–100% for viability (1, 3, and 5 days); 10% supernatants for gene expression at 7 and 14 days	Biodentine increased DMP1 expression after 14 days, whereas CEM produced comparatively limited DSPP and DMP1 responses under the evaluated conditions.	[138]
MTA-enriched PCL/chitosan scaffold	Human dental pulp stem cells	Composite scaffolds containing 30 wt% MTA relative to polymer weight; angiogenic medium; molecular analysis after 7 days	Increased VEGFR-2, Tie2, and angiopoietin-1 gene and protein expression. These responses concern the complete composite scaffold, not unmodified MTA cement.	[130]

Abbreviations: BMP-2, bone morphogenetic protein 2; DMP1, dentin matrix protein 1; DSPP, dentin sialophosphoprotein; ELISA, enzyme-linked immunosorbent assay; ERK, extracellular signal-regulated kinase; FGF4, fibroblast growth factor 4; LPS, lipopolysaccharide; PCL, polycaprolactone; TGF-β, transforming growth factor beta. Methodological note. The presented findings represent formulation- and model-specific experimental observations. Changes in gene expression, protein abundance, enzyme activity, cell survival, or mineral deposition should not be considered interchangeable endpoints. Demonstration of a molecular response does not establish organized dentin–pulp tissue regeneration, recovery of physiological pulp function, or long-term clinical success. Findings obtained with composite scaffolds should not be attributed to the cement component alone.

## Data Availability

No new data were created or analyzed in this study. Data sharing is not applicable to this article.

## Supplementary Materials

The following supporting information can be downloaded at: https://www.mdpi.com/article/10.3390/ijms272110042/s1.
