# Supplementary material for: Selected Calcium Silicate-Based Endodontic Cements: From Physicochemical Properties and Molecular Bioactivity to Indication-Specific Clinical Performance
Source: Int J Mol Sci. 2026 Nov 9;27(21):10042. doi: 10.3390/ijms272110042 (PMC13764137; doi:10.3390/ijms272110042)
Supplement: Supplementary file 1 [file ijms-27-10042-s001.zip › ijms-4669557-supplementary.pdf]

Supplementary Table S1

Methods and statistical reporting for the physicochemical data in Table 1

Reference numbers correspond to the main manuscript. Sample sizes refer to each material and experimental condition unless otherwise stated; repeated readings are distinguished from independent specimens. Values apply to the named formulations only. The ± symbol denotes SD only where explicitly identified. Differences in conditioning, media, normalization methods, and assay endpoints preclude direct numerical ranking.

Abbreviations: SD, standard deviation; RH, relative humidity; DW, distilled water; DIW, deionized water; HBSS, Hank's balanced salt solution; PBS, phosphate-buffered saline; micro-CT, micro-computed tomography; SEM, scanning electron microscopy. Means without dispersion and graphical values are identified in the relevant records.

Ref.	Material or formulation	Sample size, preparation, test conditions, and statistical reporting
**[18]**	CEM (radiopacity summary only)	Secondary report of CEM radiopacity: the value 2.227 mm Al is summarized from Torabzadeh et al. (2012). Sample size, imaging conditions, and dispersion are not established by the cited review and should not be inferred. Setting time and film thickness of the experimental NEC precursor are documented separately under the primary Asgary et al. (2008) study.
**[30]**	ProRoot MTA, Biodentine, and ERRM Fast	n = 15 per assay, mean ± SD. Setting: 10 × 1 mm gypsum moulds at 37°C/95% RH, 100-g/2-mm indenter at 5-min intervals, termed final setting by the authors. Radiopacity: 8 × 1 mm discs conditioned to final set at 37°C/95% RH, 60 kV/2 mA/0.08 s at 10 cm.
**[31]**	ERRM Putty	Initial and final setting: five parallel measurements per material; ERRM Putty initial 61.8 ± 2.5 min and final 208.0 ± 10.0 min. Plaster moulds (10 mm diameter × 2 mm height) were prewetted for 24 h at 37°C; filled moulds were kept in a water bath at 37°C. Gillmore needles: initial 100 g/2.0 mm tip; final 400 g/1.0 mm tip, applied every 5 min until no complete circular depression was produced. Open porosity: at least five measurements per condition after 7 or 28 d in DW at 37°C, vacuum/kerosene Archimedes method with drying at 105°C. The source does not explicitly define the ± terms for these outcomes; porosity is method-specific and differs from micro-CT total porosity.
**[40]**	Biodentine	radiopacity, n = 3 discs (10 × 1 mm), immediately immersed in gelatinized HBSS; means 4.1 and 3.3 mm Al after 1 and 28 d, respectively (SD shown graphically, not tabulated). X-rays: 65 ± 5 kV, 10 mA, 0.80 s, 30 cm. Final setting: 10 × 8 × 5 mm specimens in gelatinized HBSS at 37°C, 400-g Vicat indenter; 45 min is the reported graphical value; n and dispersion are not specified for this endpoint.
**[41]**	White ProRoot MTA and Biodentine	n = 13 per assay; discs 8 × 1.6 mm. Calcium release: fresh discs in 10 mL DIW at 37°C, medium replaced at each endpoint; the source specifies mean ± SD for cumulative release but does not explicitly define ± for the individual time-point values. Solubility/apparent porosity: preconditioning at 37°C/99% RH for 250 min (MTA) or 9 min (Biodentine), then 24 h in 20 mL DW and drying to stable mass. These preconditioning periods are not independently measured final setting times. Solubility is normalized to final dry mass; dispersion for solubility/porosity is not explicitly defined in the source table.
**[42]**	ProRoot MTA and Biodentine	n = 5; discs 4 × 2 mm, conditioned for 24 h at 37°C, then immersed in 10 mL DIW at 37°C for 7 d; calcium measured by ICP-OES. The numerical ± terms are retained as reported; the article does not explicitly identify their statistical definition.
**[49]**	Experimental NEC (CEM precursor) and tooth-colored ProRoot MTA	Primary report, Asgary et al. (2008). Working time, setting time, dimensional change, film thickness, and flow: ISO 6876:2001, mean ± SD. Methods states three measurements per material; Table 2 labels NEC n = 5 and MTA n = 3, so the reported counts are not fully consistent. Detailed conditioning for these tests is not specified. Setting time: NEC 50 ± 7.5 min; working time: NEC 4.5 ± 0.77 min. Film thickness was converted from µm to mm: NEC 174 ± 25 µm = 0.174 ± 0.025 mm and ProRoot MTA 452 ± 63 µm = 0.452 ± 0.063 mm. These values concern the experimental NEC, not every commercial CEM formulation.

[50]	White ProRoot MTA and Biodentine	setting n = 6 (10 × 1 mm gypsum moulds, 37°C/95% RH, 100-g/2-mm needle; termed final set by the authors); radiopacity n = 10 (10 × 1 mm, 24 h maturation, 60 kV/7 mA/0.12 s, 21 cm). Solubility n = 12 per medium/time point (20 × 1.6 mm, 24 h maturation at 37°C/95% RH, then 160 mL double-distilled water at 37°C; medium renewed daily, then weekly); percentage initial-mass loss after drying, with the long endpoint at 28 d. Solubility is mean ± SD; the source does not separately define ± for setting time or radiopacity.
[51]	Unmodified MTA Angelus	Initial 10.3 ± 1.5 min and final 18.0 ± 1.0 min; n = 3, mean ± SD. Specimens: 10 mm diameter × 1 mm height; 37 ± 1°C/98% RH. Gillmore needles: initial 113.4 g and final 453.6 g, applied every 60 s until no indentation was observed. Materials were mixed according to the manufacturer's instructions. The original article contains an inconsistency between its abstract and Table 2 regarding the assignment of initial setting times to MTA Angelus and AGM MTA. The values presented here follow Table 2 and the Discussion of the original publication.
[52]	White MTA Angelus and Biodentine	Mean ± SD; final setting n = 5 (10 × 2 mm, 37°C/95% RH, 456-g needle); radiopacity n = 5 (10 × 1 mm, 48 h conditioning, 70 kV/7 mA/0.32 s, 33 cm); pH n = 10 (10-mm-long, 1-mm-internal-diameter tubes in 10 mL DW at 37°C, water replaced between readings).
[53]	Unmodified White MTA Angelus and Biodentine	setting n = 10 per material, 10 × 5 mm specimens, 37 ± 2°C/95% RH, final Gillmore needle 453.6 g. Setting-time ± terms are not explicitly defined, although the article labels SD for other outcomes; those definitions should not automatically be transferred to setting time.
[54]	Hand-mixed CEM (Yektazist Dandan)	Primary report, Shahi et al. (2015): hand-mixed CEM (Yektazist Dandan), powder/liquid ratio 3:1. Setting n = 6, 15 × 5 mm moulds at 37°C/95–100% RH, Vicat indentation; 00:52:50 ± 00:00:36 converted to 52.83 ± 0.60 min. Film thickness: three test repetitions, 150 N applied at 3 min, thickness read at 10 min; 1.3 ± 0.05 mm as reported. Both are mean ± SD; the repetition count follows the primary Methods.
[59]	ProRoot MTA and Biodentine	the marginal-gap analysis used only one tooth per material, selected after a 25-d bacterial-leakage experiment; 3-mm pulp dressing, SEM at ×2000. Mean ± SD describe the reported gap measurements; the number of independent SEM measurements is not specified. The 15 teeth per group in the leakage experiment are not the sample size for SEM.
[60]	NeoMTA and Biodentine	n = 13 teeth per material; 5-mm apical plugs, 7 d at 37°C/100% RH, SEM at 1 and 3 mm from the apex; mean ± SD. Eight measurements were used per section.
[61]	White ProRoot MTA and Biodentine	n = 8, 5 × 5 mm cylinders assessed after 24 h by micro-CT (9.6-µm voxel); total porosity. The source does not explicitly define the ± terms.
[62]	Gray ProRoot MTA and Biodentine	n = 10 extracted teeth per material, 3-mm root-end cavities; fluid filtration at 1 d, 1 week, and 1 month after setting. Five readings over 10 min were averaged per tooth and converted to µL/min; values are mean ± SD, not linear dye penetration.
[64]	MTA Angelus and Biodentine	n = 10 teeth per material/bur subgroup; carbide and diamond bur preparations are reported separately. Dye extraction used 72 h in 2% methylene blue at 37°C, followed by 72 h in 65% nitric acid; absorbance at 550 nm, mean ± SD. Values follow the material-specific source table.
[67]	Unmodified Biodentine	solubility n = 6, 7.75 × 1.5 mm discs, 24 h maturation at 37°C/95% RH, then 30 d in 7.5 mL DIW at 37°C; percentage mass loss after drying to stable mass. Porosity n = 6, 7 × 4 mm discs, initial total porosity after 24 h maturation, micro-CT (9-µm voxel). Both outcomes are mean ± SD.
[68]	Conventional CEM (BioniqueDent, Tehran, Iran) without zinc oxide nanoparticles	Setting time: n = 6, mean ± SD; initial 11.0 ± 0.0 min and final (reported as secondary) 45.0 ± 6.64 min. Powder/liquid ratio: 1 g/0.33 mL; Plexiglas rings, 10 mm internal diameter × 1 mm thickness; initial incubation for 150 ± 1 s at 37°C/100% RH. Vicat needles: initial 100 ± 0.5 g/2 ± 0.1 mm diameter; final 456 ± 0.5 g/1 ± 0.1 mm diameter. Measurements every 3 min for the first 30 min, every 5 min up to 90 min, then every 15 min; endpoint: no impression at three separate points. pH n = 10, mean ± SD; 2 or 7 d maturation at 37°C/100% RH followed by immersion in 10 mL DIW (initial pH 6.5), with measurements after 5 or 24 h of immersion. These are two separate time variables. Solubility: n = 10, mean ± SD; 7 × 1 mm discs matured for 2 or 7 d at 37°C/100% RH, desiccated for 1 h before initial weighing, immersed in DIW at 37°C for 15 h, and desiccated again for 2 d before final weighing. Percentage loss of initial dry mass was calculated as (W1 − W2)/W1 × 100, where W1 and W2 are the dry masses before and after immersion, respectively.
[69]	ProRoot MTA	radiopacity n = 3, mean ± SD (10 × 1 mm discs, 24 h at 37°C/95% RH; 70 kV/7 mA, 30 cm). pH n = 5, fresh material in 10-mm-long, 1-mm-internal-diameter tubes in 10 mL DW, renewed between readings; the listed pH values are means without numerical dispersion.
[70]	White ProRoot MTA and Biodentine	n = 5 per assay, mean ± SD; 8 × 1.6 mm discs conditioned for 24 h at 37°C/99% RH. pH/calcium: 10 mL DIW (initial pH 6.6) at 37°C, replaced at each reading. Solubility: 24 h in 20 mL DW followed by drying at 37°C and gravimetric measurement.
[71]	ProRoot MTA and Biodentine	n = 10 acrylic teeth per material, 3-mm apical plugs in 10 mL PBS at 37°C, mean ± SD. The early values follow the source table's "3 h" label; the Methods lists the first reading at 24 h, an unresolved internal timing discrepancy.
[72]	CPM MTA mixed with DW	n = 7 premolar roots; apical and coronal openings sealed, roots in 1 mL DW at 37°C/100% RH for 7 d; calcium diffusion through dentin measured by atomic absorption spectrophotometry, mean ± SD. This is not a direct cement-disc release assay.
[73]	CEM mixed with 100%	5 × 2 mm tubes in 10 mL DIW, renewed at each endpoint; pH and calcium are mean ± SD. Independent n for these assays is not specified; the

	manufacturer-supplied liquid (1 g/0.54 mL)	microhardness sample size does not apply. Calcium units follow the source table (mg/L), whereas the Methods inconsistently uses mg/dL.
**[74]**	ProRoot MTA and Biodentine	n = 10, mean ± SD; 10 × 1 mm discs conditioned for 24 h at 37°C/95% RH, radiography at 70 kV/8 mA/0.32 s and 30 cm; five repeated images per specimen.
**[77]**	Uncontaminated Biodentine	n = 5, initial setting time 30.00 ± 0.00 min (mean ± SD), 12 × 2 mm discs at room temperature, 100-g/2-mm Gillmore needle every 5 min; this is not a final-setting measurement.
**[78]**	ProRoot MTA	Mean ± SD; solubility, three pairs of specimens (six discs, 20 × 2 mm), 7 d maturation at 37°C/100% RH followed by 24 h in 50 mL DW at 37°C, gravimetric residue method. Film thickness: three repetitions, 150 N at 3 min, measurement at 10 min. pH: 10 × 1 mm discs matured for 24 h at 37°C, then 10 mL HBSS; 7-d reading, three repeat measurements, independent specimen count not stated.
**[79]**	White ProRoot MTA, unmodified Biodentine, and ERRM	n = 10 per material in the DW control condition; 10 × 2 mm discs matured for 24 h at 37°C/100% RH and immersed for a separate 24 h in DW (pH 7.4). Listed values are mean percentage loss of initial mass; numerical dispersion for the percentage endpoint is not reported. The ERRM consistency is not specified.
**[80]**	ProRoot MTA	n = 6 per condition; 20 × 1.5 mm discs, 24 h maturation at 37°C, then DW for 24 h or 28 d; mean ± SD. The published solubility equation omits mass normalization and has an ambiguous sign; values are reproduced as reported and should not be treated as a standardized mass-loss estimate.
**[81]**	Unmodified Biodentine control without bromelain	n = 10 per assay, mean ± SD. Solubility: 20 × 2 mm discs after final set, 24 h in 25 mL DIW at 37°C, followed by desiccation; the published percentage formula uses final mass as denominator. Radiopacity: 10 × 1 mm discs after setting, 65 kVp/7 mA/0.20 s, 32 cm.
**[82]**	MTA Angelus, 1 g powder/0.330 mL water	20 specimens per material in total; the count allocated to each time point is not explicitly stated. Discs 7.75 × 1.5 mm, 24 h maturation at 37°C/100% RH, then 7 or 30 d in 7.5 mL DIW at 37°C and 24 h drying. Values are means; dispersion is not reported.
**[83]**	Original experimental MTA (Loma Linda University), not a named commercial ProRoot formulation	pH measured during setting using a temperature-compensated electrode; 12.5 refers to 3 h after mixing. The pH specimen count and dispersion are not reported.
**[84]**	Unmodified Biodentine	n = 6, mean ± SD; 4-mm-internal-diameter × 5-mm-high moulds, then 10 mL DIW at 37°C after setting; static medium without replacement.
**[85]**	MTA in non-etched 3-mm root-end cavities	80 roots across four materials and etched/non-etched subgroups; per-subgroup n and MTA brand are not stated in the abstract. One week in PBS preceded a separate week of methylene-blue exposure; linear leakage, mean ± SD.
**[86]**	MTA Angelus, Biodentine, and CEM	n = 22 teeth per material; 3-mm intraorifice barriers, 48 h at 37°C/100% RH followed by 7 d in India ink under a 75-Torr vacuum; maximum linear dye penetration. Numerical ± terms are not explicitly defined.
**[87]**	Unmodified MTA and MTA with chitosan nanohydrogel	n = 15 teeth per group, mean ± SD. The unmodified group gave 0.8333 ± 0.21882 and the modified group 0.7147 ± 0.20842. The abstract identifies spectrophotometric dye absorption, but measurement units, exposure duration, detailed dye protocol, and MTA manufacturer are not reported; these values cannot be classified as 1-week linear leakage in mm.
**[88]**	MTA Angelus, Biodentine, and ERRM (lot BP11001)	n = 19 molars per material; furcation repairs conditioned for 72 h at 37°C/100% RH beneath moist cotton and Cavit, followed by micro-CT total-porosity measurement; mean ± SD.
